# A potent and protective human neutralizing antibody targeting a novel vulnerable site of Epstein-Barr virus

**DOI:** 10.1038/s41467-021-26912-6

**Published:** 2021-11-16

**Authors:** Qian-Ying Zhu, Sisi Shan, Jinfang Yu, Si-Ying Peng, Cong Sun, Yanan Zuo, Lan-Yi Zhong, Shu-Mei Yan, Xiao Zhang, Ziqing Yang, Yong-Jian Peng, Xuanling Shi, Su-Mei Cao, Xinquan Wang, Mu-Sheng Zeng, Linqi Zhang

**Affiliations:** 1 State Key Laboratory of Oncology in South China, Collaborative Innovation Center for Cancer Medicine, Guangdong Key Laboratory of Nasopharyngeal Carcinoma Diagnosis and Therapy, Sun Yat-sen University Cancer Center (SYSUCC), 510060 Guangzhou, China; 2 Department of Laboratory Medicine, The Eighth Affiliated Hospital, Sun Yat-sen University, Shenzhen, 518003 PR China; 3 NexVac Research Center, Comprehensive AIDS Research Center, Center for Infectious Diseases Research, Beijing Advanced Innovation Center for Structural Biology, School of Medicine, Tsinghua University, 100084 Beijing, China; 4 The Ministry of Education Key Laboratory of Protein Science, Beijing Advanced Innovation Center for Structural Biology, Beijing Frontier Research Center for Biological Structure, Collaborative Innovation Center for Biotherapy, School of Life Sciences, Tsinghua University, 100084 Beijing, China; 5 Bejing IDMO Company Limited, Beijing, China; 6 State Key Laboratory of Oncology in South China, Department of Cancer Prevention Research, Sun Yat-sen University Cancer Center (SYSUCC), 510060 Guangzhou, China; 7 Institute of Biopharmaceutical and Health Engineering, Tsinghua Shenzhen International Graduate School, Tsinghua University, 518055 Shenzhen, China; 8 Institute of Biomedical Health Technology and Engineering, Shenzhen Bay Laboratory, 518132 Shenzhen, China

**Keywords:** Herpes virus, Infection, Viral infection

## Abstract

Epstein-Barr virus (EBV) is associated with a range of epithelial and B cell malignancies as well as autoimmune disorders, for which there are still no specific treatments or effective vaccines. Here, we isolate EBV gH/gL-specific antibodies from an EBV-infected individual. One antibody, 1D8, efficiently neutralizes EBV infection of two major target cell types, B cells and epithelial cells. In humanized mice, 1D8 provides protection against a high-dose EBV challenge by substantially reducing viral loads and associated tumor burden. Crystal structure analysis reveals that 1D8 binds to a key vulnerable interface between the D-I/D-II domains of the viral gH/gL protein, especially the D-II of the gH, thereby interfering with the gH/gL-mediated membrane fusion and binding to target cells. Overall, we identify a potent and protective neutralizing antibody capable of reducing the EBV load. The novel vulnerable site represents an attractive target that is potentially important for antibody and vaccine intervention against EBV infection.

## Abstract (web summary)

Currently, there are no treatments or vaccines against Epstein-Barr virus (EBV) infection. Here, Zhu et al. isolate gH/gL-specific antibodies from infected donors and identify 1D8, that substantially reduces infection levels in both, B- and epithelial cells, and reduces tumor burdens in EBV-challanged humanized mice due to interferance with the gH/gL-mediated membrane fusion and binding.

## Introduction

Epstein-Barr virus (EBV) is associated with a wide range of diseases in humans such as infectious mononucleosis and lymphoproliferative disorders, as well as epithelial and B cell malignancies including nasopharyngeal carcinoma and Burkitt’s lymphoma^1–5^. Despite decades of research, a safe and effective vaccine against EBV still remains elusive, largely due to a lack of knowledge regarding the specificity and magnitude of immune responses required for protection^6–10^. EBV-infected individuals produce broad and potent neutralizing antibodies that can inhibit infection of both epithelial cells and B cells in vitro^11–15^. However, their specificity to viral antigens and potential mechanism of neutralization are not clear.

Recent studies on monoclonal antibodies (mAbs) revealed some of the intricate interactions between antibodies and viral surface antigens, providing critical insights into the potential targets for antibody neutralization and vaccine development^16–20^. The reported mAbs recognize exclusively viral surface glycoproteins that work in concert in determining viral tropism and mediating viral fusion with the target cells, such as gp350, gH/gL, gB, and gp42^21–24^. Recently, gH/gL and gB, which together constitute the fusion machinery of EBV, have drawn increasing attention as newer generations of antibodies targeting this machinery demonstrate broad and potent inhibitory activity against EBV infection of both B cells and epithelial cells^25,26^, as well as cross-neutralizing reactivity to related herpesviruses of non-human primate^11,27,28^.

As components of the fusion machinery, gH/gL and gB demonstrate unique structural and functional features that are critical for viral entry, but they also inadvertently expose some vulnerable sites during the process, and become susceptible to antibody binding and neutralization^29–31^. The gH/gL protein consists of four distinct domains named domain-I (D-I) to domain-IV (D-IV), forming an elongated structure^32^. D-I is formed by gL and the N-terminus of gH, while D-II to D-IV are formed by the rest of gH. D-I and D-II are connected through a linker helix and form a structurally distinct groove. For viral fusion to occur, gH/gL must interact with gB, which triggers a cascade of events involving dramatic structural changes of gB from the pre- to the post-fusion conformation^30,31^. Mutations in the D-I and D-I/D-II interfaces of gH/gL were shown to affect the membrane fusion process, suggesting that these regions of gH/gL are important for the interaction and activation of gB^33,34^.

Apart from the fusion machinery, EBV infection requires additional surface glycoproteins to complete the entry process, but the accessory molecules involved are rather different between B cells and epithelial cells^35^. For instance, EBV utilizes gp350, one of the most abundant glycoproteins on the viral envelope, to attach to the cell surface through high-affinity interaction with CD21 or CD35 in B cells infection^36–38^. Such attachment promotes the bridging effect of another surface glycoprotein, gp42, which inserts itself between gH/gL and human leukocyte antigen (HLA) class II, to trigger the downstream fusion machinery^39,40^. Interestingly, gp42 has an inhibitory effect on epithelial cell infection, suggesting a different entry mechanism in B cells and epithelial cells^41^. For infection of epithelial cells, gH/gL first binds to integrin and NMHC-IIA^42^ on the cell surface. The fusion machinery then interacts with neuropilin 1 (NRP1) and ephrin receptor A2 (EphA2)^43–45^, which leads to a conformational transition of gB, facilitating viral fusion^30,46^.

Most of the current anti-gH/gL antibodies are of murine origin^39,47^. E1D1, CL59, and CL40 can block epithelial cell infection but fail to efficiently neutralize B cell infection^24^. A human neutralizing antibody targeting gH/gL, AMMO1, was recently isolated from an EBV-infected individual^24^. AMMO1 can potently block infection of both B cells and epithelial cells in vitro. AMMO1 can also protect humanized mice from EBV challenge and provide sterilizing immunity in macaques against oral challenge with rhesus lymphocryptovirus, the EBV homologue that infects rhesus macaques^28^. These findings indicate that a vaccine capable of inducing AMMO1-like neutralizing antibodies may protect human from EBV infection. Cryo-electron microscopy (cryoEM) analysis of the AMMO1-gH/gL-gp42 complex revealed that AMMO1 binds to an epitope between D-I and D-II of gH/gL^24^, which serves as a more precise target for future vaccine design and development. Recently, another human neutralizing antibody 769B10 was identified and found to compete for binding to gH/gL with AMMO1 and CL40, although no structural information was available^11^.

In this work, we seek to isolate more neutralizing antibodies from EBV-infected individuals targeting the EBV gH/gL. After screening a large number of infected individuals, we successfully isolated the anti-gH/gL-antibody 1D8, which is capable of efficiently neutralizing EBV infection of epithelial cells and B cells in vitro. 1D8 also provides protection against EBV challenge in humanized mice by significantly reducing the viral loads and associated tumor burden. Using X-ray crystallography, we determine the structure of the 1D8-gH/gL complex and show that 1D8 recognizes a novel epitope located at the top of the groove between D-I and D-II of gH/gL, especially the D-II of gH. Notably, this epitope is located on the opposite side of that recognized by AMMO1, CL40, and 769B10. In addition, 1D8 also significantly inhibit viral membrane fusion and gH/gL binding to epithelial cell receptor EphA2. We believe that this new vulnerable site, together with that recognized by AMMO1, CL40, and 769B10, suggests that D-I and D-II represent an attractive target that is potentially important for antibody and vaccine intervention against EBV infection.

## Results

### Isolation of human monoclonal antibodies targeting the EBV gH/gL

**Fig. 1:**
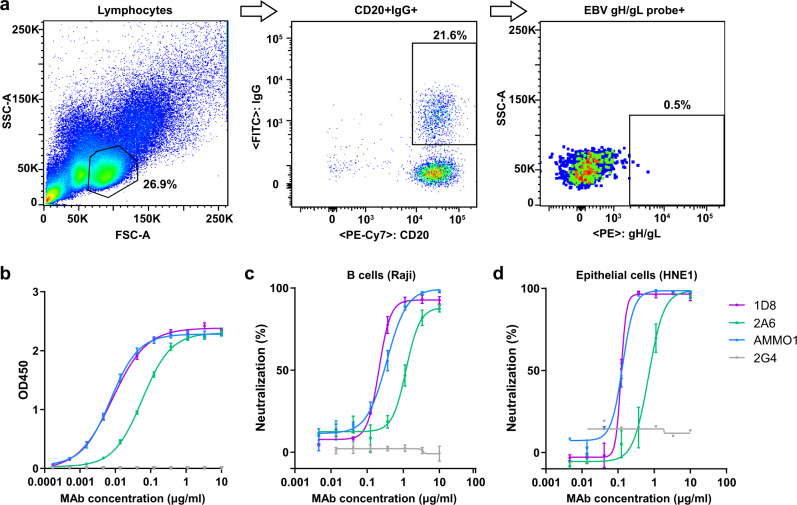
Isolation of gH/gL-specific monoclonal antibodies using single B cell sorting and cloning. **a** FACS-based sorting strategy for gH/gL-specific B cells. **b** Binding activities of 1D8 and 2A6, the positive control AMMO1, and the negative control 2G4 to EBV gH/gL measured by ELISA. The data are presented as means ± SEM from two replicates. **c** Neutralizing activities of 1D8 and 2A6, the positive control AMMO1, and the negative control 2G4 against EBV infection of Raji B cell lines and (**d**) HNE1 epithelial cell line. The data shown are means ± SEM from two replicates. SSC-A, side-scatter area; FSC-A, forward-scatter area. Source data are provided as a Source Data File.

We first screened plasma samples from a cohort of high-risk individuals and nasopharyngeal carcinoma patients^48,49^ for those with the highest levels of binding and neutralizing activity. Of the 48 plasma samples screened, donor 27 from the high-risk group had antibodies with the highest affinity for gH/gL measured by ELISA, with the half-maximal effective concentration (EC_50_) corresponding to a 6874-fold dilution (Supplementary Fig. 1a). The same plasma sample also displayed the most potent neutralizing activity against EBV infection of HNE1 epithelial cells and Raji B cells, with respective half-maximal inhibitory concentrations (IC_50_) corresponding to a 273-fold and 1250-fold dilution (Supplementary Fig. 1b). To isolate monoclonal antibodies, we used phycoerythrin (PE) conjugated gH/gL as baits to stain and sort the antigen-specific memory B cells from the peripheral blood mononuclear cells (PBMCs) of donor 27 using flow cytometry (Fig. 1a). Out of a total 54 sorted single B cells, we were able to clone and express 10 full-length human immunoglobulin G1 (IgG1) genes in transfected 293T cells. Two antibodies, 1D8 and 2A6, were found to have strong binding to gH/gL. As shown in Fig. 1b, 1D8 showed ~7-fold stronger binding to gH/gL than 2A6, with concentration for 50% of maximal effect (EC_50_) of 0.008 μg/ml and 0.057 μg/ml, respectively. 1D8 also demonstrated higher neutralizing activity than 2A6 against EBV infection of HNE1 epithelial cells and Raji B cells (Fig. 1c, d and Supplementary Table 1). The IC_50_ of 1D8 was about 6-fold lower than that of 2A6 for both cell types. Notably, 1D8 displayed comparable binding and neutralizing activities to AMMO1, a potent gH/gL-specific neutralizing antibody previously isolated from an EBV-infected individual^24^. The equilibrium dissociation constant (KD) measured by surface plasmon resonance (SPR) was 0.59 nM for 1D8 and 0.14 nM for AMMO1 (Supplementary Fig. 1c, d and Supplementary Table 2). When tested for neutralizing activity against EBV infection, 1D8 and AMMO1 showed IC_50_ values of 0.238 μg/ml and 0.318 μg/ml in Raji B cells, as well as 0.123 μg/ml and 0.127 μg/ml in HNE1 epithelial cells, respectively (Fig. 1c, d and Supplementary Table 1). Notably, neither 1D8 nor AMMO1 could completely block EBV infection in vitro. There were approximately 8% and 2% of Raji B cells, as well as 6% and 2% of HNE1 epithelial cells, were infected after treatment with 10 μg/ml of 1D8 and AMMO1, respectively (Fig. 1c, d). For neutralization in primary B cells, the IC_50_ of 1D8 and AMMO1 are 0.361 μg/ml and 0.227 μg/ml, respectively. In Bmi1-immortalized nasopharyngeal epithelial cell line (NPEC1-Bmi1)^42^, the IC_50_ of 1D8 and AMMO1 are 0.173 μg/ml and ~10 μg/ml, respectively.

### 1D8 protects against lethal EBV challenge in humanized mice

**Fig. 2:**
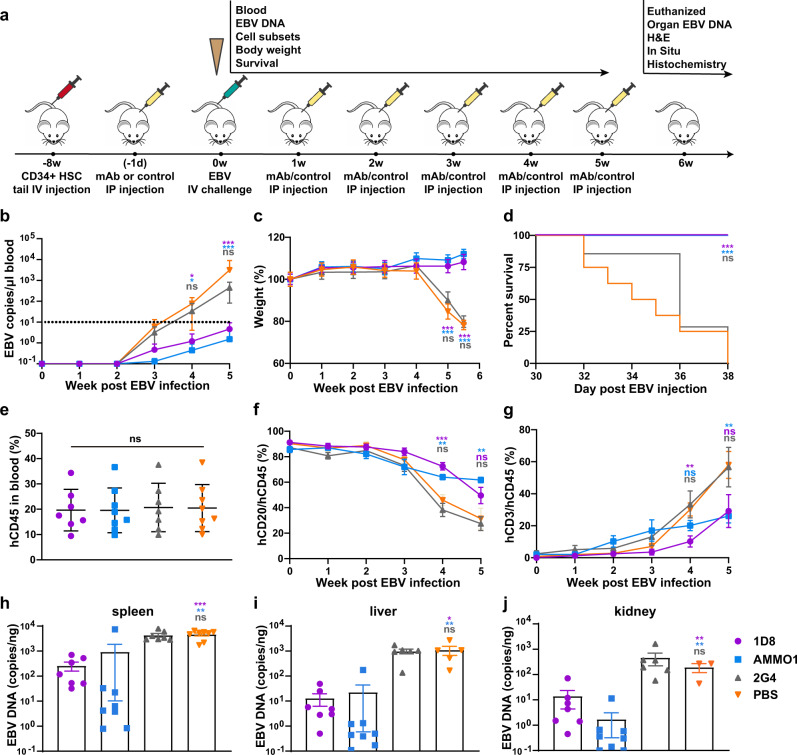
Protective efficacy of 1D8 against lethal EBV challenge in humanized mice. **a** Timeline for engrafting CD34 + human hematopoietic stem cells (HSC), antibody administration, viral challenge, and monitoring for various biological and clinical outcomes. 400 μg of 1D8 (*n* = 7), positive control AMMO1 (*n* = 8), negative control 2G4 (*n* = 7), or PBS (*n* = 8) were administered to the humanized mice via intraperitoneal injection either 24 h prior to or weekly for 5 weeks after intravenous challenge with Akata EBV. **b** EBV DNA in the peripheral blood, (**c**) body weight, and (**d**) survival were monitored weekly. The percent changes in (**e**) hCD45 + , (**f**) hCD20 + , or (**g**) hCD3+ cells over the experiment period. On week 6 post infection, (**h**) virus titers in spleen, (**i**) liver, (**j**) kidney were analyzed. All data are presented as mean ± SEM. *p < 0.05; **p < 0.01; ***p < 0.001; ns, no significant, two-tailed unpaired Student’s *t* test. (**b**) 1D8 vs PBS in 4w *p = 0.017, AMMO1 vs PBS in 4w *p = 0.010, ***p < 0.0001; (**c**) ***p < 0.0001; (**d**) 1D8 vs PBS ***p = 0.0002, AMMO1 vs PBS ***p = 0.0004, log-rank test (Mantel-Cox); (**f**) 1D8 vs PBS in 4w ***p = 0.0003, AMMO1 vs PBS in 4w **p = 0.0021, AMMO1 vs PBS in 5w **p = 0.001; (**g**) 1D8 vs PBS in 4w **p = 0.0028, AMMO1 vs PBS in 5w **p = 0.0034; (**h**) ***p < 0.0001, **p = 0.0048; (**i**) *p = 0.0126, **p = 0.008; (**j**) 1D8 vs PBS **p = 0.0052, AMMO1 vs PBS **p = 0.0013. Source data are provided as a Source Data File.

To test the protective potential of 1D8 in vivo, we used a humanized mouse model reconstituted with human cord blood-derived CD34+ stem cells that became susceptible to EBV infection and disease after approximately 8 weeks of development and maturation^50–52^. The entire experimental protocol and assays conducted to evaluate protection are outlined in Fig. 2a. Briefly, we administrated 400 μg of 1D8, AMMO1 as positive control, or 2G4^53^ and PBS as negative controls to groups of seven to eight humanized mice via the intraperitoneal (i.p.) route. On the following day, the animals were challenged with 1000 50% transforming dose (TD_50_) Akata EBV via the intravenous (i.v.) route. In the ensuing up to 6-week period, all animals received the testing antibodies or PBS weekly via the i.p. route and were monitored for body weight, survival, as well as various virologic and immunologic parameters.

EBV DNA in the peripheral blood measured by quantitative PCR reflected the distinctions in clinical manifestation between these animals (Fig. 2b). In the animals treated with 1D8 and AMMO1, EBV DNA became detectable on week 3 after challenge and slowly increased in the following two weeks, but no animals had EBV DNA copy numbers greater than 10 copies/μl blood at week 5 post-challenge. By contrast, in the animals treated with 2G4 and PBS, EBV DNA rapidly increased from week 3 onwards after the challenge and reached about 100-fold higher copy numbers than 1D8 and AMMO1 treated animals at week 5 post-challenge (Fig. 2b). All animals in the 1D8 and AMMO1 treated groups survived the challenge and demonstrated relatively stable body weight without obvious pathology (Fig. 2c, d). By contrast, negative control animals (2G4 and PBS groups) began significantly losing weight starting on day 28 (4 weeks), succumbed to disease and had to be euthanized by day 38 after the challenge.

By the time they were ready for the protection experiments, the reconstituted animals had about 20% human CD45+ lymphocytes in the peripheral blood, nearly 90% of which were human CD20+ B cells and <1% were human CD3+ T cells (Fig. 2e–g). In addition, the dynamic change of human CD20+CD45+ B cells and CD3+CD45+ T cells in the peripheral blood was correlated with distinct pattern of disease progression between these animals. Animals in the 1D8 and AMMO1 treated groups showed a relatively slower decrease in the percentage of CD20+CD45+ B cells compared with the 2G4 and PBS treated groups. As a result, the slower increase in percentage of CD3+CD45+ T cells was noticed in this group of animals compared to those treated by 2G4 and PBS (Fig. 2f, g). Of note, these cells are unlikely virus-specific but mainly represent the process of reconstitution^54^.

Furthermore, EBV DNA copy numbers measured in the spleen, liver and kidney collected at necropsy shared a similar trend with those measured in the peripheral blood (Fig. 2h–j). The copy numbers of EBV DNA were significantly lower in the 1D8 and AMMO1 treated groups than in the 2G4 and PBS treated groups, although the copy numbers were generally higher in the spleen than in the liver and kidney (Fig. 2h–j).

We also conducted a similar experiment in humanized mice to test whether a single dose of antibody could reduce the burden of tumors caused by EBV infection. As shown in Supplementary Fig. 2a, we administrated 400 μg of antibodies or PBS to humanized mice. The next day, animals were challenged with 1,000 TD_50_ Akata EBV. All animals were monitored for body weight, survival, as well as virologic parameters up to 7 weeks. In the 1D8 and AMMO1 treated groups, EBV DNA in peripheral blood was significantly lower than that in the control groups (Supplementary Fig. 2b). Meanwhile, all animals in 1D8 and AMMO1 treated groups survived the challenge, maintained stable body weight and had lower spleen weight collected at necropsy (Supplementary Fig. 2c–e). Furthermore, EBV DNA copy numbers measured in spleen, liver, and kidney were also lower in 1D8 than the control groups, and in AMMO1 than the control groups. However, comparing between AMMO1 and 1D8 treated groups, the EBV levels were significantly lower only in the spleen while quite comparable in the liver and kidney (Supplementary Fig. 2f–h).

Taken together, these results demonstrated that 1D8 as well as the positive control AMMO1 might reduce EBV replication and, to a certain extent, provide protection from a lethal EBV challenge.

### Marked reduction in viral replication and tissue damages in the protected animals

**Fig. 3:**
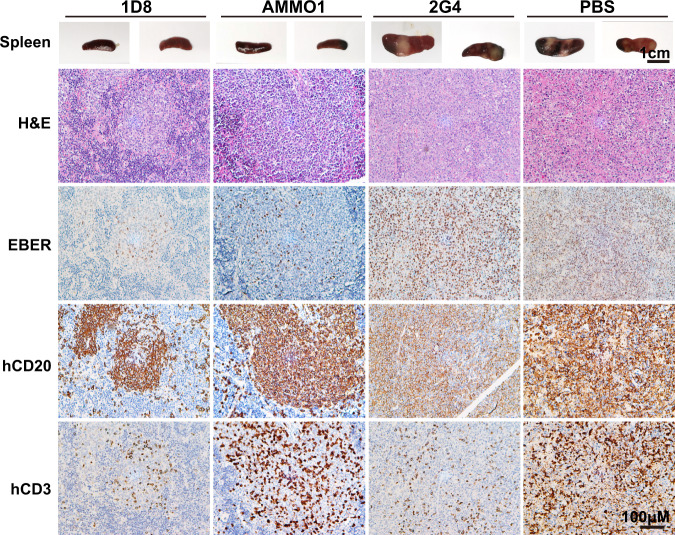
1D8 reduces viral replication and tissue damages in humanized mice. Representative of macroscopic spleens and splenic sections stained for hematoxylin and eosin (H&E), EBV encoded RNA (EBER), human CD20 (hCD20), and human CD3 (hCD3) at necropsy. The scale bars are indicated. Each image is representative of a group of 7–8 mice.

To study the impact of protection at the tissue levels, we collected the spleen, liver and kidney of the animals at the necropsy. The most profound and visible changes were observed in the spleens. Morphologically, the spleens from the 2G4 and PBS groups were clearly enlarged with a few irregular and pale tumors across the entire surface. By contrast, the spleens in the 1D8 and AMMO1 groups were normal in size and color, without visible tumors (Fig. 3). We went on to perform histopathology analysis on the spleen sections using hematoxylin and eosin (H&E) staining, immunohistochemistry (IHC) for hCD3 and hCD20, as well as in situ hybridization for Epstein-Barr virus-encoded RNAs (EBERs) (Fig. 3). All mice treated with 2G4 or PBS presented with typically large B-cell lymphomas in the white pulp regions, which were positive for hCD20 and EBER. They were abundant and widely distributed across the tissue sections. Morphologically their proliferations destroyed the underlying architecture of the tissue with some infiltration by hCD3+ T cells. Additionally, areas of coagulative necrosis were often present in the spleens of mice from the 2G4 and PBS groups. By contrast, in the 1D8 and AMMO1 groups, the overall tissue architecture remained largely intact, even if some atypical large transformed cells could also be seen. Among the large number of hCD20+ B cells in the white pulp areas, EBER+ cells were relatively fewer compared to the PBS and 2G4 treated groups. A number of hCD3+ T cells were also found scattered within. Similarly, in the hepatic and renal sections from 2G4 and PBS groups, a large number of hCD20+ and EBER+ B cells were identified, while they were rare in the 1D8 and AMMO1 groups (Supplementary Fig. 3). The infected cells were frequently found near the blood vessels in both the liver and kidney, likely the results of migration and seeding from the blood circulation. Collectively, these results show that 1D8 and AMMO1 can significantly reduce viral replication and tissue damage relative to 2G4 and PBS, offering an explanation for their in vivo protection against a lethal EBV challenge.

### 1D8 binds to a novel epitope on gH/gL

**Fig. 4:**
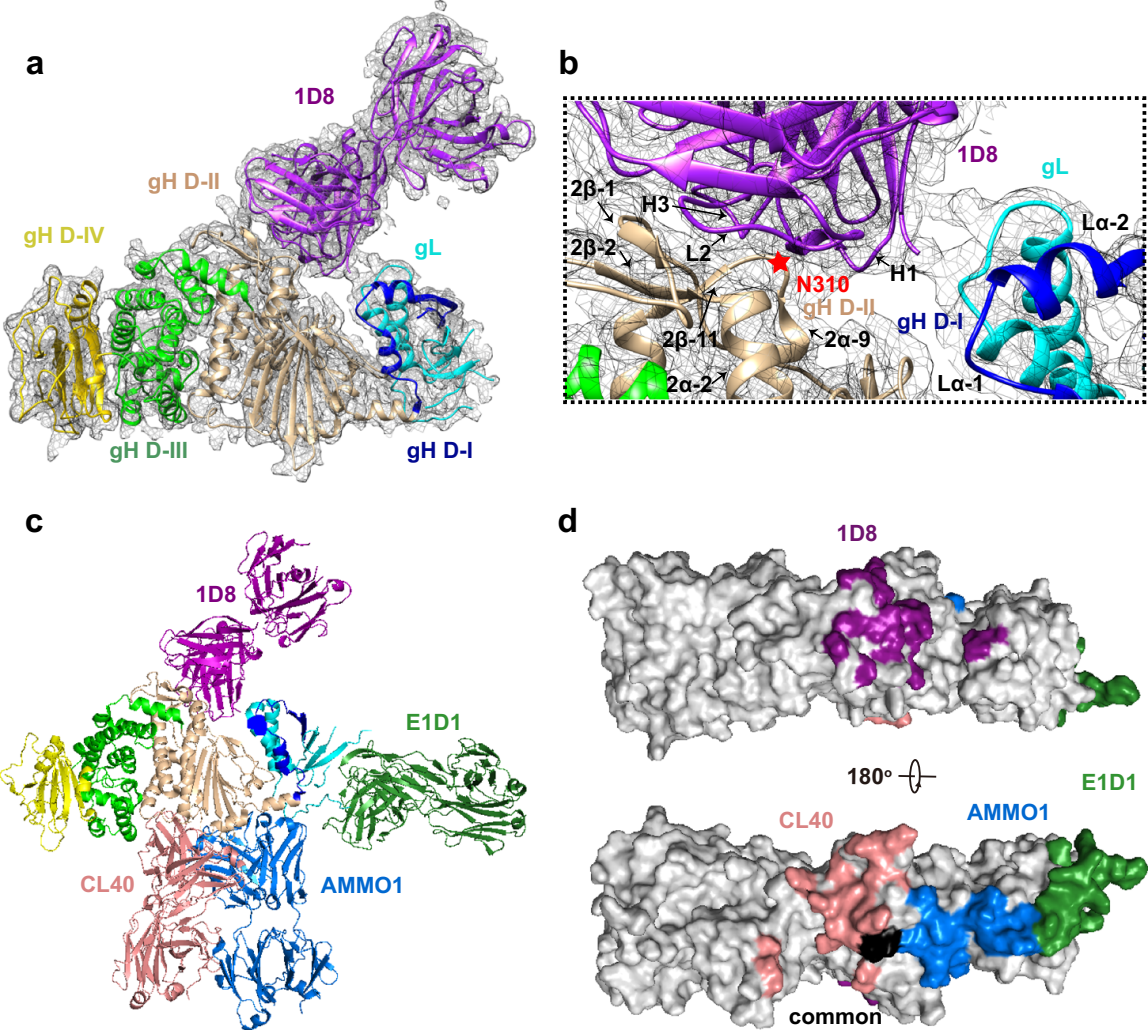
1D8 targets a novel epitope on gH/gL. **a** Structure overview in a cartoon representation with gL in cyan, gH D-I in blue, D-II in wheat, D-III in green, D-IV in yellow, and 1D8 Fab in purple. The map is contoured at 1.2 RMS to show the density. **b** Zoomed-in view of the interaction between 1D8 and D-I and D-II. The key binding residue N310 of gH was indicated by a red star. **c** Cartoon representation of Fab 1D8 and other previously published Fabs AMMO1, CL40, and E1D1 bound to a single gH/gL molecule. The color scheme for gH/gL is as in (**a**) whereas 1D8 Fab in purple, AMMO1 Fab in light blue, CL40 Fab in pink, and E1D1 Fab in dark green. **d** Surface mapping of the four Fab epitopes on gH/gL with the same color in (**c**). Areas in black indicate the region where structural change was found upon binding to AMMO1 or CL40.

To understand the neutralizing mechanism of 1D8, we sought to determine the structure of the 1D8-gH/gL complex by X-ray crystallography. After screening nearly 100 crystals with relatively weak diffraction, we obtained a dataset with 4.2 angstrom resolution and solved the structure by molecular replacement (Supplementary Table 3). 1D8 was found to bind to the interface at the top of the groove formed by D-I and D-II of gH/gL, especially the D-II of gH (Fig. 4a). While CDRL1, CDRL3 and CDRH2 made negligible contribution, CDRL2, CDRH1 and CDRH3 interacted substantially with gH and gL. CDRL1 interacted with gH largely through 2α-2 and 2β-2 whereas CDRH1 and CDRH3 through the loop between 2α-9 and 2β-11. CDRH3 also bound to the loop between 2α-1 and 2β-1 of gH. The heavy chain of 1D8 was also found to interact with gL through the loop between Lα-1 and Lα-2 (Fig. 4b). To study the locality and topology of 1D8 recognition relative to that of AMMO1, we went further to collect ~5000 cryo-EM images of the gH/gL bound to the 1D8 Fab and the AMMO1 Fab (Supplementary Fig. 4a). While the data was inadequate to obtain 3D density map for model building, we managed to build a 2D model of the gH/gL-1D8-AMMO1 ternary complex (Supplementary Fig. 4b) by superimposing the crystal structure of gH/gL-1D8 onto the previously reported gH/gL-gp42-AMMO1 complexes^24^. The calculated projection of the gH/gL-1D8-AMMO1 model matched well with the 2D average of gH/gL-1D8-AMMO1 single-particle cryo-EM data (Supplementary Fig. 4a, b). This result further validated the structural features of the gH/gL-1D8 crystal structure and demonstrated that epitope of the 1D8 is distinct from that AMMO1. Of note, as the 4.2 angstrom resolution was insufficient to determine the side chain interaction at the interface, the above interpretations need to be taken with caution.

Furthermore, to identify the key residue for antibody binding, we generated a series of single alanine substitutions for the contacting residues on gH/gL. As 4.2 angstrom resolution is not high enough to resolve the side chains, we increased the distance cutoff from 4 to 6 angstrom to include all potential contacting residues. Except for two (L122A and C312A), the remaining 27 mutants were successfully expressed in the supernatant of transfected 293F cells. We then performed ELISA to assess the impact of these mutants on 1D8 binding. One mutant, N310A, located in the loop between 2α-9 and 2β-11 of gH, was identified to specifically reduce the binding of 1D8 but not AMMO1 (Fig. 4b and Supplementary Fig. 5a, b). When measured by SPR, the binding affinity of 1D8 to this mutant dropped to 31.6 nM, representing more than 53-fold decrease from the 0.59 nM of the wild type gH/gL. However, no significant change of binding was found for AMMO1 compared to the wild type gH/gL (0.14 nM vs. 0.19 nM) (Supplementary Fig. 5c, d and Supplementary Table 2). We are uncertain why only N310A resulted in substantial reduction in binding while the impacts of the remaining 26 mutants were rather minimal. It is possible that multiple mutations are required to work in concert to effectively disrupt 1D8 recognition, given its potent binding activity of 0.59 nM to gH/gL measured by SPR. Alternatively, as the structure resolution was relatively low, our predication on the epitope residues were not as entirely precise as expected. Further studies would be warranted to resolve this uncertainty.

The unique binding mode of 1D8 is further supported by superimposing the antibodies with known structural information onto the same gH/gL molecule. As shown in Fig. 4c, d, 1D8 binds to gH/gL at the top of the groove formed between D-I and D-II, especially the D-II of gH, while AMMO1 binds to the opposite side of the molecule through a discontinuous epitope formed at the D-I/D-II interface. The mouse-derived antibody CL40 partially overlaps with the epitope of AMMO1 by binding to an epitope on gH at the interface between D-II and D-III^24^. A recently isolated human mAb 769B10 also competed for gH/gL binding with AMMO1 and CL40, indicating a shared vulnerable site for neutralization^11^. Another mouse antibody E1D1, however, only recognizes gL^39,47^ (Fig. 4c, d). Lastly, we used bio-layer interferometry (BLI) to confirm that 1D8 does not compete with any of these antibodies in binding to gH/gL (Supplementary Fig. 5e). Collectively, these results indicate that 1D8 recognizes a novel vulnerable site on gH/gL and provide a good rationale for the combined use with other antibodies to improve inhibition of EBV infection.

### 1D8 inhibits gH/gL-mediated membrane fusion and binding to B and epithelial cells

**Fig. 5:**
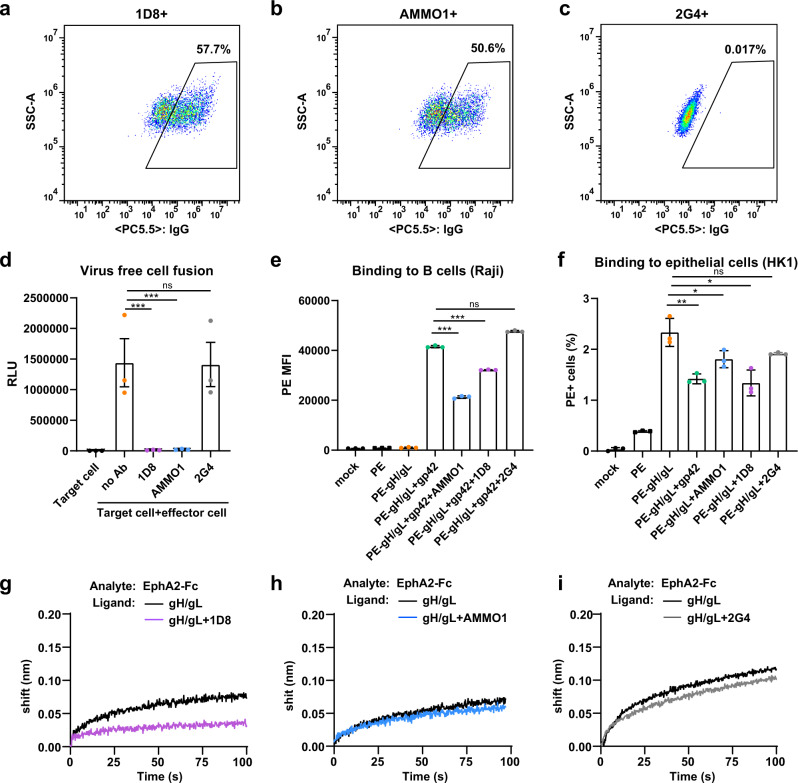
1D8 interferes with cell fusion and binding. Quality control of gH/gL expression on the surface of CHO-K1 cells by staining with 1D8 (**a**), positive control AMMO1 (**b**), and negative control 2G4 (**c**) before proceeded to the fusion experiment shown in (**d**). **d** Marked reduction in cell-cell fusion in the presence of 1D8, the positive control AMMMO1, but not the negative control 2G4. Marked reduction in gH/gL binding to Raji B cell (**e**) and HK1 epithelial cell (**f**) in the presence of 1D8, the positive control AMMO1, but not the negative control 2G4. Binding of EphA1-Fc to gH/gL was reduced by 1D8 (**g**), but not by AMMO1 (**h**) or 2G4 (**i**) measured by BLI. All data are presented as mean ± SEM from three replicates. *p < 0.05; **p < 0.01; ***p < 0.001; ns, no significant; two-tailed unpaired Student’s *t* test. **d** ***p < 0.0001; (**e**) ***p < 0.0001; (**f**) gp42 **p = 0.0057, AMMO1 *p = 0.0478, 1D8 *p = 0.010. SSC-A, side-scatter area; PC5.5, PerCP-Cy5.5; RLU, relative light unit; SA-PE, streptavidin-phycoerythrin; MFI, mean fluorescence intensity. Source data are provided as a Source Data File.

We next studied the ability of 1D8 to inhibit gH/gL-mediated membrane fusion by monitoring the fusion efficiency between the effector and target cells. Specifically, effector CHO-K1 cells expressing the gH/gL and gB fusion machinery were incubated with a saturated concentration of 1D8 or relevant controls at 37 °C for 1 h before mixing at a 1:1 ratio with the target HEK293 cells. The inhibitory activity was measured 24 h afterwards via the luciferase activity in the cell lysates, which only became detectable when fusion occurred. As shown in Fig. 5a–c, the effector CHO-K1 cells expressed good levels of gH/gL as measured by flow cytometry. In the presence of 1D8 or AMMO1, the fusion activity was barely measurable and similar to the background where only the effector cells were present (Fig. 5d). By contrast, incubation with the negative controls 2G4 or PBS resulted in high levels of luciferase activity beyond one million relative light unit (RLU). In addition, we tested the ability of 1D8 to interfere with the binding of fluorescently labeled gH/gL to Raji B cells and HK1 epithelial cells, both of which are susceptible to EBV infection in vitro^55,56^. Gp42 was included in the assessment of the binding to B cells but not for the epithelial cells, since the gH/gL-gp42 complex is specifically required for B cell activation and fusion^39^. 1D8 and relevant controls were incubated with fluorescent-labeled gH/gL at 37 °C for 1 h before mixing with B cells or epithelial cells and further incubation on ice for 1 h. The levels of inhibition of gH/gL-mediated binding to both cell types were measured by flow cytometry. As shown in Fig. 5e, pre-incubation with 1D8 and AMMO1 significantly reduced but did not completely abrogate gH/gL-gp42 binding to B cells. While no difference was found between the negative controls 2G4 and PBS, AMMO1 appeared to be more potent than 1D8 in interfering with the binding of gH/gL to B cells. Conversely, 1D8 seems to be more powerful than AMMO1 in inhibiting the binding of gH/gL to the epithelial cells, whereas the negative controls showed negligible effect (Fig. 5f). Lastly, we studied the ability of 1D8 to inhibit the interaction between gH/gL and EphA2, a recently identified receptor for EBV infection of epithelial cells that depends on an interaction with gH/gL^43,44^. Consistent with an earlier report^24^, the interaction between EphA2-Fc and gH/gL was indeed rather weak as measured by BLI. Nevertheless, pre-incubation of gH/gL with 1D8 did result in a small and clear reduction in the interaction between gH/gL and EphA2 (Fig. 5g). Conversely, such an effect was not noticed for AMMO1 or 2G4 (Fig. 5h, i). This may explain why 1D8 was more effective than AMMO1 in inhibiting EBV infection of epithelial cells (see above), although the underlying mechanism warrants further investigation. Taken together, these findings indicate that 1D8 as well as the positive control AMMO1 can significantly inhibit gH/gL-mediated membrane fusion and binding to B and epithelial cells, either through direct blocking of binding or by sterically interfering with the downstream interactions required for EBV infection.

## Discussion

Neutralizing antibodies exert their function by targeting vulnerable sites on the viral envelope glycoproteins. Identifying the neutralizing mAbs and their recognized epitopes is therefore the first crucial step for understanding the protective antibody response, which can inform the rational development of antibody-based therapy and vaccines^19,20^. In some EBV-infected individuals, high levels of serum neutralizing antibodies have been identified capable of blocking infection of both B cells and epithelial cells^11^. This finding indicates that the human immune system can generate potent neutralizing antibodies to clear the infection and/or attenuate disease progression. However, the antigen and epitope specificity, as well as the potential mechanism of neutralization of these antibodies are not entirely clear.

We report here the isolation and characterization of the human neutralizing antibody 1D8, which substantially reduces infection of B cells and epithelial cells in vitro. Passive delivery of 1D8 significantly reduced the viral loads and tumor burden of EBV-induced lymphoma in humanized mice. Structural analysis of the 1D8-gH/gL complex identified a novel epitope at the top groove of gH/gL between D-I and D-II, especially the D-II of gH, which is distinct from any of the reported antibodies. In addition, 1D8 was found to inhibit viral membrane fusion and reduce the binding of gH/gL to the epithelial cell receptor EphA2^43,44^. We believe that this new vulnerable site, together with that recognized by AMMO1, CL40, and 769B10, suggests that D-I and D-II represent an attractive target that is potentially important for antibody and vaccine intervention against EBV infection.

A couple of points need to be highlighted here. First, as both B cells and epithelial cells are major targets for EBV infection^57^, it is highly desirable to isolate neutralizing antibodies capable of blocking the virus and protecting both cell types from infection. 1D8, together with recently reported AMMO1^24^ and 769B10^11^, are three representatives of this class of human antibodies with dual tropism. However, we are uncertain how much this type of antibodies contributes to the overall neutralizing activities in the infected individuals. Given the low frequency in identifying 1D8, AMMO1 and 769B10 antibodies among the isolated memory B cells^24^, it is reasonable to assume they are quite rare and might only be induced among a small proportion of naturally infected patients. Compared to gp350, gH/gL is much less abundant and therefore has a quantitative disadvantage in immune recognition and stimulation^21^. However, identification of 1D8, AMMO1, and 769B10 epitopes around D-I and D-II offer an unprecedented opportunity to expose this vulnerable site in much more precise and persistent manner so that more focused and stronger immune response like 1D8, AMMO1, and 769B10 could be generated. This could be done either by including gH/gL in the vaccine regimen^11,25^ or singling out D-I and D-II domain as epitope-focused immunogens. Both approaches would require careful design and validation to ensure proper structure and exposure of vulnerable sites recognized by like 1D8, AMMO1, and 769B10. In support of this notion, nanoparticles displaying gH/gL elicited a strong neutralizing antibody response against EBV infection of both target cell types^11^, even if this exciting report requires further confirmation. Lastly, given the relatively conserved nature of this region among herpesviruses^27,30^, carefully designed D-I and D-II immunogens may be able to induce an even broader and stronger cross-neutralizing antibody response against a wide variety of viral strains.

**Fig. 6:**
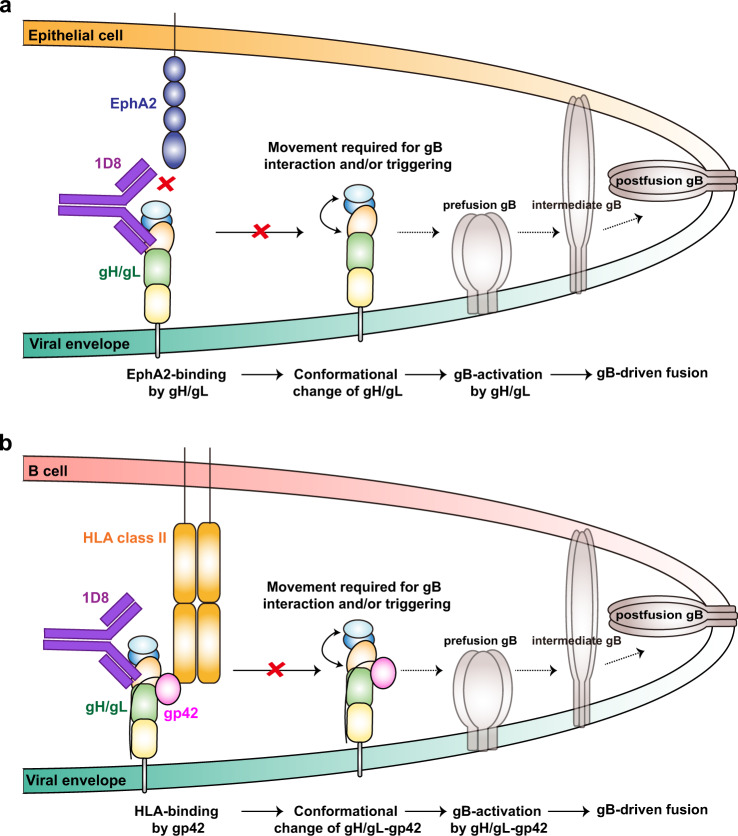
Possible mechanisms of 1D8-mediated neutralization. **a** For epithelial cells, 1D8 could interfere the interaction between gH/gL and EphA2 either by directly restricting access to the interface or by indirectly posing allosteric hindrance. It could also restrict the movement across the D-I/D-II groove of gH/gL that is required for gB interaction and triggering. **b** For B cells, 1D8 could also restrict the movement across the D-I/D-II groove of gH/gL that is required for downstream viral entry. 1D8 does not appear influence interaction between gp42 and its receptor HLA class II.

Second, despite structural and functional insights, we are still uncertain of the exact mechanism through which 1D8 neutralizes EBV infection of both target cell types. Structurally, although 4.2 angstrom resolution was insufficient to determine the atomic interaction at the interface, 1D8 recognized a novel epitope within the groove between D-I and D-II, especially the D-II of gH. AMMO1, on the other hand, was found to bind a discontinuous epitope spanning D-I and D-II on the opposite ridge of the groove^24^ whereas 769B10 was found to compete with AMMO1^11^. Such convergence on D-I and D-II domains suggests a common mechanism of neutralization, either by affecting coordination within and across D-I and D-II or their interaction with other viral glycoproteins such as gB or gp42 required for downstream viral entry (Fig. 6a, b). AMMO1 was postulated to lock D-I, D-II and the linker helix, preventing proper movement required for interaction and activation of gB^58^. As residues with the D-I and D-II groove also mediate membrane fusion and several of these critical residues are near the epitope of 1D8^33,34,58,59^, it stands to reason that 1D8 could also exert its neutralization activity by inhibiting the fusion process. Instead of acting like a molecular clamp as AMMO1, 1D8 may act more like a molecular wedge forcing into the space within the groove. Certainly, as 1D8 and AMMO1 and 769B10 bind distinct epitopes, there must be some differences in the exact mechanisms underlying their inhibitory effects. For example, AMMO1 appears to be more potent than 1D8 in interfering with the binding of gH/gL to B cells (Fig. 5e), perhaps due to its ability to displace the c-terminal domain of gp42 through the gp42 N173 glycan^24^. Conversely, 1D8 seems to be more powerful than AMMO1 in inhibiting binding of gH/gL to epithelial cells, likely by affecting the interaction between EphA2-Fc and gH/gL (Figs. 5f, g and 6a). In any case, the 1D8 antibody identified in this study represents another potent human neutralizing antibody that can be used alone or in combination with other antibodies such as AMMO1 and 769B10 for antibody-based interventions against EBV infection. The epitope defined here will also assist the rational design of vaccines focusing more on the vulnerable sites to elicit powerful neutralizing antibodies like 1D8, AMMO1, and 769B10.

## Methods

### Ethical standards

The utilization of human samples was approved by the Ethics Review Committee of the Sun Yat-sen University Cancer Center (SYSUCC, YP2009051) and conducted in accordance with the Declaration of Helsinki. Written informed consents forms were signed by all human research participants. All animal experiments with infectious EBV were performed in the animal biosafety level 2 facilities at Sun Yat-sen University Zhongshan School of Medicine, which was approved by the Committee on the Ethics of Animal Experiments of Sun Yat-sen University (SYSUCC, L102012019070D). The animal studies were carried out in strict accordance with the recommendations promulgated in the Guide for the Care and Use of Laboratory Animals of the Ministry of Science and Technology of the People’s Republic of China.

### Human subjects

We collected plasma samples from 48 participants including 23 histologically diagnosed NPC cases and 25 non-NPC high-risk healthy controls in a screening program in Sihui County in Guangdong Province of China from 2007 and 2018. Peripheral blood mononuclear cell (PBMC) sample of donors 27 were collected in 2018. The screening program has been introduced in detail in other manuscript^60^. Primary B cells were isolated from healthy donor PBMC using the CD19 MicroBead Kit (Miltenyi Biotec Cat#130-117-034).

### Cell lines

All cell lines were cultured at 37 °C in a humidified atmosphere comprising 5% CO_2_. 293T cells (ATCC) were grown in DMEM (GIBCO Cat#C11995500BT) + 10% FBS (GIBCO Cat#10099141). CHO K-1 cells (ATCC) were maintained in Ham’s F-12 (GIBCO Cat#11765054) +10% FBS. Raji cells (ATCC), HNE1 cells^61^ and HK1 cells^62^ were maintained in RMPI1640 + 10% FBS. Akata B cells^63^ harboring a modified EBV, in which the thymidine kinase gene has been replaced with a neomycin and green fluorescence protein (GFP) cassette (Akata-GFP), were grown in RMPI 1640 (GIBCO Cat#C11875500BT) + 5% FBS. 293F cells (ThermoFisher) were maintained in Freestyle 293 medium (Union Cat#UP1000) with gentle shaking. Bmi1-immortalized nasopharyngeal epithelial cells (NPEC1-Bmi1) were cultured in keratinocyte serum-free medium (17005-075; Invitrogen, California). All cells were grown with 100 U/ml pencillin and 100 μg/ml streptomycin.

### Humanized mice

The construction of the humanized mice was based on NOD*.Cg-Prkdc*^*em1IDMO*^*Il2rg*^*em2IDMO*^ mice (NOD-*Prkdc*^null^ *IL2R*γ^null^, NPI^®^)^64^, which were kept in a specific pathogen free (SPF) facility and obtained from BEIJING IDMO Co., Ltd. Animals were maintained under standard conditions of humidity (50%), temperature (25 ± 2 °C) in a 12 h light and 12 h dark cycle. To generate the humanized immune system, mice were i.p. injected with a single dose of Busulfan at 20 mg/kg body weight. After 48 h post-injection, the mice received an intravenous tail injection of human CD34 + cells, which were isolated from umbilical cord blood (Beijing Novay biotech) with a purity of over 90%. Human CD45+ cells in peripheral blood of each humanized mouse were detected at 4 and 8 weeks post engraftment by flow cytometry.

### Plasmids

The gH/gL [residues 19 to 679 of gH and residues 24 to 137 of gL were linked by (G4S)_3_] and gp42 (residue 34 to 223) fragments were amplified from the bacterial artificial chromosome (BAC) of EBV-M81 by PCR and cloned into the pcDNA3.1 plasmid with an N-terminal CD5 leader peptide and a C-terminal HIS Tag. Targeted mutations were introduced into pcDNA3.1-CD5-gH/gL using the ClonExpress MultiS One Step Cloning Kit (Vazyme Cat# C113-02) and were confirmed by Sanger sequencing. The pCAGGS expression plasmids for gH, gL, gB, and pT7EMCLuc (which carries a luciferase-containing reporter plasmid under the control of the T7 promoter) were kindly provided by Dr. R. Longnecker. EphA2 (27–534) fused with a C-terminal human IgG1-Fc domain by a (GGGGS)_3_ linker was cloned into a pcDNA3.1 vector.

### Recombinant antibody cloning

The VH and VK/Vλ genes of reference antibodies CL40, E1D1 and AMMO1 were obtained from PDB and codon optimized genes were synthesized by Tsingke Biological Technology Company. Antibody heavy chain and light chain variable gene fragments were obtained using separate primer pairs^65^ with restriction enzyme cutting sites, including VH primers with 5’AgeI and 3’SalI, VK primers with 5’AgeI and 3’BsiWI, and Vλ primers with 5’AgeI and 3’XhoI. Then PCR products were cloned into antibody expression vectors containing the constant regions of human IgG1. The sequences of the recombinant plasmids were verified by Sanger sequencing.

### Recombinant protein expression

The 293F cells were transfected with plasmids encoding EBV glycoproteins, EphA2-Fc and recombinant antibodies at a density of 1.5 × 10^6^ cells/ml in Freestyle 293 medium using PEI (Polysciences Cat#24765-1) transfection reagent according to the manufacturer’s instructions. After five days, the culture supernatant containing EBV glycoprotein was collected and passed through Ni-NTA resin (GE Healthcare Cat#17-3712-02), followed by washing (PBS with 20 mM imidazole, pH 7.4) and elution (PBS with 250 mM imidazole, pH 7.4). The proteins were further purified by size exclusion chromatography (SEC) and dialyzed into PBS. Clarified cell supernatant containing recombinant antibodies or EphA2-Fc was passed over Protein A agarose (GenScript Cat#L00210), followed by extensive washing with PBS, and then eluted with 10 mL of 0.3 M glycine, pH 2.0 into 1 mL of 1 M Tris HCl, pH 8.0. Purified proteins were then dialyzed into PBS.

### Recombinant protein biotinylation

gH/gL were biotinylated at a theoretical 1.5:1 biotin/protein ratio using the EZ-Link Sulfo-NHS-Biotin (ThermoFisher Cat#21338) at room temperature for 30 min. Free biotin was removed by 3 successive rounds of dilution with PBS.

### Preparation of the antigen-binding fragment

1D8 Fab was obtained by digesting 1D8 IgG with Endoproteinase Lys-C (Sigma Cat#11420429001) at 37 °C (1 mg IgG: 250 ng Lys C) for 12 h. Fab fragments were isolated with Fc fragments using protein A agarose, then further purified by SEC.

### Bio-layer interferometry

Antibody competition binding assays (Octet Red 96, ForteBio, Pall LLC): 250 nM gH/gL was captured onto HIS1K sensors (ForteBio, Pall LLC) for 120 s. The baseline interference was then read for 60 s in KB buffer (PBS, 0.1% BSA, 0.02%Tween). Then the sensor was loaded with 1D8 (10 μg/ml) or KB (blank) for 60 s and balanced in KB for 60 s, followed by association with 10 μg/ml competitive antibodies (1D8, AMMO1, CL40, E1D1) for 120 s and association with KB for 120 s. One gH/gL-1D8 loaded sensor was immersed in buffer as a reference during the association and dissociation steps and used to subtract the background signal. Antibody/EphA2 competition binding assays: 2 μg/ml gH/gL-biotin was immobilized on streptavidin biosensors (ForteBio, Pall LLC, Cat# 18-5019), and then immersed into KB for 60 s. Then the sensor was loaded with 1D8 (50 nM), AMMO1 (50 nM), 2G4 (50 nM) or KB (blank) for 60 s and balanced in KB for 60 s, followed by associated with 1000 nM EphA2-Fc for 100 s and association with KB for 120 s. One gH/gL-antibody loaded sensor was immersed in buffer as a reference during the association and dissociation steps and used to subtract the background signal.

### Surface plasmon resonance

The binding kinetics and affinity of antibodies for gH/gL or their mutants were analyzed by SPR (Biacore 8 K, GE Healthcare). Anti-human IgG (Fc) antibody was covalently immobilized onto a CM5 sensor chip (GE Healthcare Cat# BR-1005-30) via amine groups in 10 mM sodium acetate buffer (pH 5.0) for a final RU of around 5000. Specifically, antibodies 1D8 or AMMO1 (2 μg/ml) were captured by anti-human IgG antibody for 10 s. Diluted gH/gL or their mutants were run at a flow rate of 30 μl/min in HBS-EP (aqueous buffer containing 0.01 M HEPES pH 7.4, 0.15 M NaCl, 3 mM EDTA and 0.05%(v/v) Tween 20, filtered through a 0.2 μm filter). The sensograms were fit to a 1:1 binding model using the Biacore Insight Evaluation Software (GE Healthcare).

### Enzyme-linked immunosorbent assay (ELISA)

For ELISA, 100 ng/well of EBV glycoprotein was coated in 96-well enzyme-linked immunosorbent assay plates overnight at 4 °C. Then, the plates were blocked with 5% bovine serum albumin (BSA) in PBS and 0.1% Tween-20 (blocking buffer) at 37 °C for 1 h. After blocking, the plates were washed three times with 0.1% Tween-20 in PBS (washing buffer). Plasma samples or recombinant antibodies were diluted serially in blocking buffer and incubated at 37 °C for 1 h. Following three times of washing, a 1:4000 goat anti-human IgG-HRP (Promega Cat#W4031) in blocking buffer was added to each well and incubated at 37 °C for 45 min. Plates were washed five times and incubated with 3,3′,5,5′-tetramethylbenzidine substrate (TMB) (TIANGEN Cat#PA107-01) for 5 min at room temperature. Then 1 M hydrochloric acid (HCl) was added and the OD_450_ was read on a microplate reader (Epoch2).

For the binding analysis of gH/gL mutants (<4 angstrom cutoff), 500 ng/well of antibody was coated in plates overnight at 4 °C. The plates were then blocked and washed. The gH/gL mutants were diluted serially in blocking buffer and incubated at 37 °C for 1 h. Following three times of washing, 1:3000 diluted mouse anti-his antibody (TRANSGEN BIOTECH Cat#HT501-02) in blocking buffer was added to each well and incubated at 37 °C for 1 h. After three times of washing, a 1:5000 diluted goat anti-mouse-HRP antibody (Invitrogen Cat#31430) was added and incubated at 37 °C for 1 h. The final steps were the same as above. For antibody binding to various gH/gL mutants (4–6 angstrom cutoff), 293 T cells in 24-well plate were transfected with 500 ng gH/gL mutated plasmids and gH/gL protein secreted into the cell supernatants were captured by antibody-coated plates. Antibody binding and detection were conducted as routine ELISA assay.

### B cells sorting

Cryopreserved 10^7^ PBMC were thawed into 1 ml preheated RPMI1640, centrifuged at 300 × *g* for 5 min, resuspended in 500 μl FACS buffer (PBS + 2% FBS), and incubated with 200 nM his-tagged antigen (gH/gL) for 45 min at 4 °C. The PBMC were then washed two times with 1 ml FACS buffer and resuspended in 100 μl FACS buffer. The PBMC were stained with the following antibodies: CD3-PE-Cy5 (BD Biosciences Cat#555341) at a 1:25 dilution, CD14-PE-Cy5 (eBioscience Cat#15-0149-42) at a 1:50 dilution, CD16-PE-Cy5 (BD Biosciences Cat#555408) at a 1:25 dilution, CD235a-PE-Cy5 (BD Biosciences Cat#559944) at a 1:100 dilution, CD19-APC-Cy7 (BD Biosciences Cat#348794) at a 1:100 dilution, CD20-PE-Cy7 (BD Biosciences Cat#335793) at a 1:200 dilution, IgG-FITC (BD Biosciences Cat#555786) at a 1:25 dilution, and anti-his-PE (BioLegend Cat#362603) at a 1:20 dilution for 30 min at 4 °C. The PBMC were washed three times with 1 ml FACS buffer and resuspended in 500 μl FACS buffer, then subjected to FACS on a BD FACS Aria II (BD Biosciences).

Antigen-positive B cells (CD3-, CD14-, CD16-, CD235a-, CD19+, CD20+, IgG+, PE+) were sorted individually into 96-well PCR vital-plates containing 20 μl first strand buffer (5 μl first strand buffer, 0.5 μl of RNase inhibitor (Invitrogen Cat#10777019), 1.25 μl of 100 μM DTT, 0.06 μl of IGEPAL (Sigma Cat#I8896).

### VH/VL recovery from sorted cells

Wells containing sorted cells were mixed with 6 μl of reverse transcription (RT) buffer containing 1.5 μl mixed primers specific for human IgG, IgM, IgD, IgA1, IgA2, K and λ constant gene regions, 1.5 μl of 25 mM dNTP mix (Invitrogen Cat#10297117), and 0.25 μl of superscript III reverse transcriptase (Invitrogen Cat#18080085). The RT temperature program included 42 °C for 10 min, 25 °C for 10 min, 60 °C for 50 min, and 94 °C for 5 min, followed by a hold at 4 °C. The VH, VK and Vλ genes were amplified from 5 μl of cDNA separately using nested PCR (HotStarTaq DNA Polymerase, QIAGEN Cat#203205). The PCR products were purified and subjected to Sanger sequencing. Then, the VH, VK and Vλ variable genes were assembled into functional linear Ig gene expression cassettes by overlap-extension PCR. The function of the expressed antibodies was determined using ELISA screening.

### Virus production

Akata cells carrying EBV, in which the thymidine kinase gene was interrupted with a double cassette expressing GFP and a neomycin resistance gene, were resuspended in FBS-free RPMI 1640 medium at a concentration of 2–3 × 10^6^ cells per ml, followed by induction with 0.75% (v/v) of goat anti-human immunoglobulin G serum (Shuangliu Zhenglong Biochem Lab Cat#H0111-6) for 6 h at 37 °C. After culture in fresh RPMI1640 medium supplemented with 5% FBS for 3 days, virus from the supernatant was collected under sterile conditions, passed through two Millipore filters (0.8 and 0.45 µm), concentrated 100-fold by high-speed centrifugation at 50,000 g, and then resuspended in fresh FBS-free RPMI1640. The virus was stored at 80 °C and thawed immediately before infection. To assess the virus titer, 10-fold dilutions of EBV were used to inoculate 2 × 10^5^ PBMC per well in 24-well plates with 2 μg/ml cyclosporin A (CsA) (Sigma Cat#C3662). The cultures were fed weekly by replacing half of the medium with fresh medium containing CsA. After 6 weeks, the TD_50_ was determined based on the number of proliferating lymphocytes in the wells^54^.

### Neutralization assay

Plasma samples from study individuals or recombinant antibodies were incubated with nearly 1,000TD_50_ GFP-expressing EBV at serial dilutions for 3 h at 4 °C. Then the mixtures were added to Raji B cells, HNE1 epithelial cells, primary B cells and NPEC1-Bmi1 and incubated for 3 h at 37 °C. Then the unbound virus was removed by washing with PBS twice. Infected cells were cultured in fresh medium for 48 h, followed by detection and analysis of GFP-positive cells using a flow cytometer and FlowJo 10 software (FlowJo, USA). The neutralization rate of each sample was defined as: (%GFP+ cells in the positive control well containing virus alone—%GFP+ cells in the plasma or antibody containing well)/%GFP+ cells in the positive control well × 100.

### EBV infection in humanized mice

At 8 weeks post CD34+ stem cells transfer, 400 μg of experimental or control antibodies were i.p. injected per humanized mouse. After 24 h, the mice received a dose of Akata EBV equivalent to 1000 TD_50_ via i.v. injection. In the following period, the mice received a dose of 0.4 mg antibody weekly. The blood collection and recording of body weight and health status were also done every week. The mice were euthanized 6 weeks post-EBV infection or earlier if they became clinically ill (e.g. body weight loss of approximately 20%). For the second animal experiment, humanized mice received a single dose of 400 μg antibody before EBV challenge. All animals were monitored for body weight, survival, as well as virological parameters up to 7 weeks.

### Detection of EBV DNA in blood and tissues

DNA was extracted from the peripheral blood (100 μl) or tissues of the mice using commercial DNA extraction kits (Omega Cat#D3392-02). The EBV genome copy numbers were determined by real-time PCR (Roche Light Cycler 480) using the TaqMan BamHI probes (sense: 5′-CCCAACACTCCACCACACC-3′; antisense: 5′-TCTTAGGAGCTGTCCGAGGG-3′). The copy numbers of EBV were quantified using a standard EBV genome (BDS biotech Cat#BDS-BW-087) as control.

### H&E staining, IHC, and in situ hybridization

Tissues were fixed in 10% formalin and embedded in paraffin. Consecutive sections were used for staining with H&E. Immunostaining of human T cells and B cells was performed using hCD3 antibody (VENTANA Cat#790-4341) and hCD20 antibody (VENTANA Cat#760–2531) at 1:200 dilution. EBERs were stained by in situ hybridization using the EBER detection kit (ZSGB-BIO Cat#ISH-7001–100), according to the manufacturer’s instructions. Histological staining was evaluated by experienced pathologists.

### Detection of human immune cells in the blood of humanized mice

Peripheral blood of mice was treated with 1 ml red blood cell lysis buffer (BioLegend Cat#420301) at room temperature for 10 min. Then the cells were centrifuged at 300 *g*, washed twice with PBS, resuspended in PBS, and stained with antibodies including anti-human CD45-PE (BD Biosciences Cat#555483), CD3-PerCP-Cy5.5 (BD Biosciences Cat#560835) and CD20-FITC (BD Biosciences Cat#555622) at 1:100 dilution for 30 min at 4 °C. After washing with PBS, the percentage of CD3+ or CD20+ cells among the CD45+ cells was quantified using a flow cytometer.

### Cell-surface binding assays

For the cell-surface binding assay, 1 mg of gH/gL-biotin conjugated with SA-PE (gH/gL-PE) was diluted in 10 ml of PBS into individual wells of a 96 well plate. An equimolar amount of gp42 was added to select wells containing gH/gL-PE. 5 mg/ml of monoclonal antibodies, including 1D8, AMMO1, or 2G4, were added to select wells containing gH/gL with or without gp42 and incubated for 1 h at 37 °C. At the same time, adherent HK1 cells were trypsinized (NCM Biotech Cat#C40100), washed with RMPI 1640 and then allowed to recover at 37 °C in a humidified atmosphere comprising 5% CO_2_ for 1 h with gentle agitation twice during the period. Recovered HK1 and Raji cells were pelleted by centrifugation at 300 *g* for 5 min and then resuspended at a density of 2 × 10^6^ cells/ml in ice cold 0.5% bovine serum albumin (BSA) in PBS. Then, 100 μl of the HK1 or Raji cells suspension were added to wells containing SA-PE, gH/gL-PE with or without gp42 and antibodies, and incubated on ice for 1 h. The cells were pelleted by centrifugation at 300 *g* for 5 min, washed with 1 ml of ice cold 0.5% BSA in PBS, pelleted again and resuspended in 10% phosphate buffered formalin. The amount of PE staining was quantified using a flow cytometer.

### Virus-free fusion assay

Effector CHO-K1 cells were transiently transfected with expression plasmid (pCAGGS-gH, pCAGGS-gL, pCAGGS-gB and pT7EMCLuc, which carries a luciferase-containing reporter plasmid under the control of the T7 promoter). Target cells (HEK-293T) were transfected with expression plasmid pCAGT7 (expressing T7 DNA polymerase). After 24 h, the effector cells were trypsinized and resuspended at a density of 1 × 10^6^ cells/ml. Aliquots comprising 250 μl/well of effector cell suspension was pre-incubated with 2 μg 1D8, AMMO1 or 2G4 antibody at 37 °C for 1 h. Then the target cells were also trypsinized and resuspended at a density of 1 × 10^6^ cells/ml. An aliquot comprising 250 μl of the effector cell suspension was added to the effector cells with or without antibody. After 24 h, the medium was aspirated and the cells were lysed in 100 μl of luciferase agent (Dual-Glo Luciferase Assay System Cat#E2940). Then, 75 μl of cell lysate was transferred to a white-bottom assay plate and luciferase activity was read on a GloMax-96 Microplate Luminometer (Promega).

### Cell-surface staining

Following 24 h after expression plasmid transfection, the effector CHO-K1 cells were trypsinized and resuspended at a density of 1 × 10^6^ cells/ml. The expression level of gH/gL was detected using the indicated antibody. AMMO1 and 1D8 were used for gH/gL staining and 2G4 as a control. Then, 10 μg/ml of antibody was added to the cell suspension and incubated at 4 °C for 1 h. The cells were washed twice with PBS and stained with human IgG-PerCP-Cy5.5 (PC5.5) (BioLegend Cat# 409312) at a 1:100 dilution. After washing with PBS, the percentage of PC5.5+ cells was quantified using a flow cytometer (CytoFLEX, BECKMAN).

### Crystallization of the 1D8 Fab and data collection

To purify the gH/gL-1D8 Fab complex, 1D8 Fab was incubated with gH/gL for 1 h on ice in HBS buffer, and the mixture was then subjected to gel filtration chromatography. Fractions containing the complex were pooled and concentrated to 10 mg/ml. Crystals were successfully grown at 18 °C in sitting drops, over wells containing 200 mM sodium citrate, 100 mM HEPES sodium salt, pH 7.5, 15 % w/v MPD. The drops were made by mixing 200 nl gH/gL-1D8 Fab complex in HBS buffer with 200 nl well solution. Crystals were harvested, soaked briefly in 200 mM sodium citrate, 100 mM HEPES sodium salt, pH 7.5, 15 % w/v MPD, 20% glycerol, and flash-frozen in liquid nitrogen. Diffraction data were collected at the BL17U beam line of the Shanghai Synchrotron Research Facility (SSRF). Diffraction data were processed with HKL2000 and the crystal diffracted to 4.2 Å. The data processing statistics are listed in Supplementary Table 3.

### Structure solution and refinement

The structure was determined via the molecular replacement method using PHASER in CCP4 suite. The search models were gH/gL (PDB code 5T1D) and the antibody with the highest sequence identity with 1D8. Density map improvement by atoms update and refinement was performed with ARP/wARP29. Subsequent model building and refinement were performed using COOT and PHENIX, respectively. Final Ramachandran statistics indicated that 91.48% residues were in favored conformations, 7.06% allowed and 1.46% outliers for the final structure. The structural refinement statistics are listed in Supplementary Table 3. All structural figures were generated with PyMol (DeLano, 2002).

### Cryo-EM sample preparation and data collection

We reconstituted the complex of the gH/gL bound to the 1D8 Fab and the AMMO1 Fab at 1:1:1 molar ratio. Aliquots of complexes (4 μl, 0.5 mg/ml, in buffer containing 20 mM Tris pH 8.0 and 150 mM NaCl) were applied to glow-discharged holey carbon grids (Quantifoil grid, Au 300 mesh, R1.2/1.3). The grids were then blotted and plunge-frozen into liquid ethane using Vitrobot Mark IV (Thermo Fisher Scientific). Images for complexes were recorded using 300 kV FEI Titan Krios microscope (Thermo Fisher Scientific) at Tsinghua University. We collect ~5000 movies for complexes at a nominal magnification of 29,000× and at a defocus range between −1.2 and −1.5 μm. Each movie has a total accumulate exposure of 50 e − /Å^2^ fractionated in 32 frames of 175 ms exposure. The final image was binned 2-fold to a pixel size of 0.97 Å. Motion Correction (MotionCor2 v.1.2.6)^66^, CTFestimation (GCTF v.1.18)^67^ and non-templated particle picking (Gautomatch v.0.56, http://www.mrc-lmb.cam.ac.uk/kzhang/) were automatically executed by TsingTitan.py program. Sequential data processing and 2D projection of gH/gL-1D8-AMMO1 model was carried out on RELION3.1^68^.

### Statistical analysis

Unless noted otherwise, a two-tailed, unpaired *t-*test was used to assess statistical significance. Statistical calculations were performed in GraphPad Prism 8. The number of replicates and a description of the statistical method are provided in the corresponding figure legends. Differences with *P* values of less than 0.05 were considered to be statistically significant. *p < 0.05, **p < 0.01, ***p < 0.001, ns=not significant.

### Reporting summary

Further information on research design is available in the Nature Research Reporting Summary linked to this article.

## Supplementary information

Supplementary Information

Reporting Summary

## Data Availability

Data generated or analyzed during this study are included in this published article (and its supplementary information files). All other data are also available from the corresponding author upon reasonable requests. The atomic model of 1D8-gH/gL (PDB ID: 7D5Z; https://www.rcsb.org/structure/7D5Z) has been deposited in the Protein Data Bank. The Sequences of 1D8 antibodies have been deposited in Genbank with accession codes OK484490(1D8-HC) and OK484491(1D8-KC). Source data are provided with this paper.

## Source data

Source Data
