# Supplementary material for: A potent and protective human neutralizing antibody targeting a novel vulnerable site of Epstein-Barr virus
Source: Nat Commun. 2021 Nov 16;12:6624. doi: 10.1038/s41467-021-26912-6 (PMC8595662; doi:10.1038/s41467-021-26912-6)
Supplement: Supplementary file 1 — Supplementary Information [file 41467_2021_26912_MOESM1_ESM.pdf]

Supplementary Information for
A potent and protective human neutralizing antibody targeting a novel vulnerable site of Epstein-Barr Virus

Qian-Ying Zhu[1,6,9], Sisi Shan[2,9], Jinfang Yu[3,9], Si-Ying Peng[4], Cong Sun[1], Yanan Zuo[2], Lan-Yi Zhong[1], Shu-Mei Yan[1], Xiao Zhang[1], Ziqing Yang[2], Yong-Jian Peng[1], Xuanling Shi[2], Su-Mei Cao[5], Xinquan Wang[3✉], Mu-Sheng Zeng[1✉], Linqi Zhang[2,7,8✉]

[1]State Key Laboratory of Oncology in South China, Collaborative Innovation Center for Cancer Medicine, Guangdong Key Laboratory of Nasopharyngeal Carcinoma Diagnosis and Therapy, Sun Yat-sen University Cancer Center (SYSUCC), Guangzhou 510060, China.
[2]NexVac Research Center, Comprehensive AIDS Research Center, Center for Infectious Diseases Research, and Beijing Advanced Innovation Center for Structural Biology, School of Medicine, Tsinghua University, Beijing 100084, China.
[3]The Ministry of Education Key Laboratory of Protein Science, Beijing Advanced Innovation Center for Structural Biology, Beijing Frontier Research Center for Biological Structure, Collaborative Innovation Center for Biotherapy, School of Life Sciences, Tsinghua University, 100084 Beijing, China.
[4]Bejing IDMO Company Limited, Beijing, China.
[5]State Key Laboratory of Oncology in South China, Department of Cancer Prevention Research, Sun Yat-sen University Cancer Center (SYSUCC), Guangzhou 510060, China.
[6]Department of Laboratory Medicine, The Eighth Affiliated Hospital, Sun Yat-sen University, Shenzhen 518003, PR China.
[7]Institute of Biopharmaceutical and Health Engineering, Tsinghua Shenzhen International Graduate School, Tsinghua University, 518055 Shenzhen, China.
[8]Institute of Biomedical Health Technology and Engineering, Shenzhen Bay Laboratory, 518132 Shenzhen, China.
[9]These authors contributed equally.
✉email:
xinquanwang@mail.tsinghua.edu.cn;
zengmsh@sysucc.org.cn;
zhanglinqi@mail.tsinghua.edu.cn

Supplementary Figures

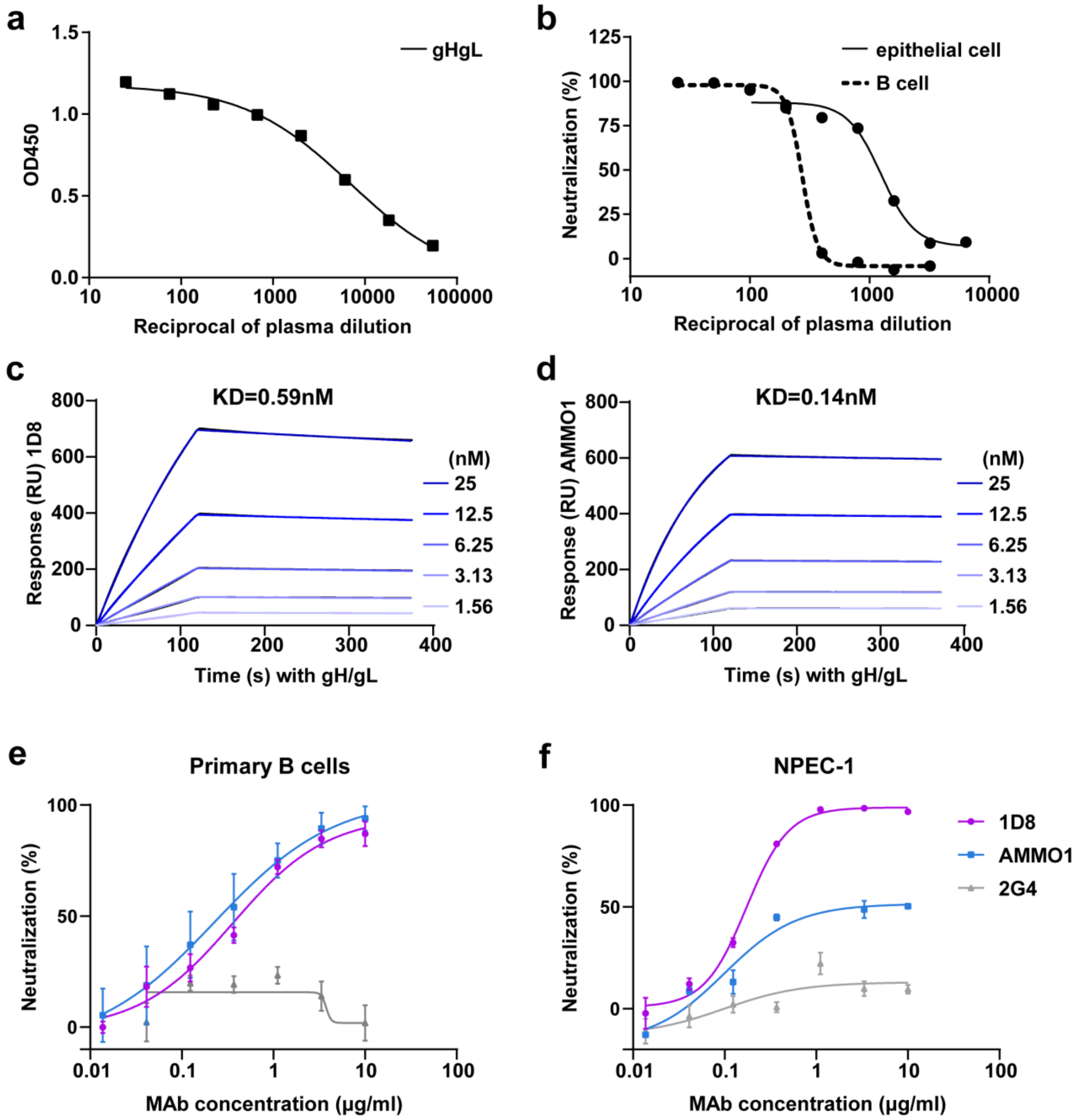

Supplementary Figure 1. Plasma binding and neutralizing activities from donor 27. (**a**) Plasma binding activities to gH/gL measured by ELISA. (**b**) Plasma neutralizing activities against EBV infection of Raji B cells and HNE1 epithelial cells. Binding activity of 1D8 (**c**) or AMMO1 (**d**) to gH/gL measured by SPR. (**e**) Neutralizing activities of 1D8, AMMO1 and 2G4 against EBV infection of primary B cells and (**f**) Bmi1-immortalized nasopharyngeal epithelial cell line (NPEC1-Bmi1). The data shown is means ± SEM from three replicates. Source

data are provided as a Source Data File.

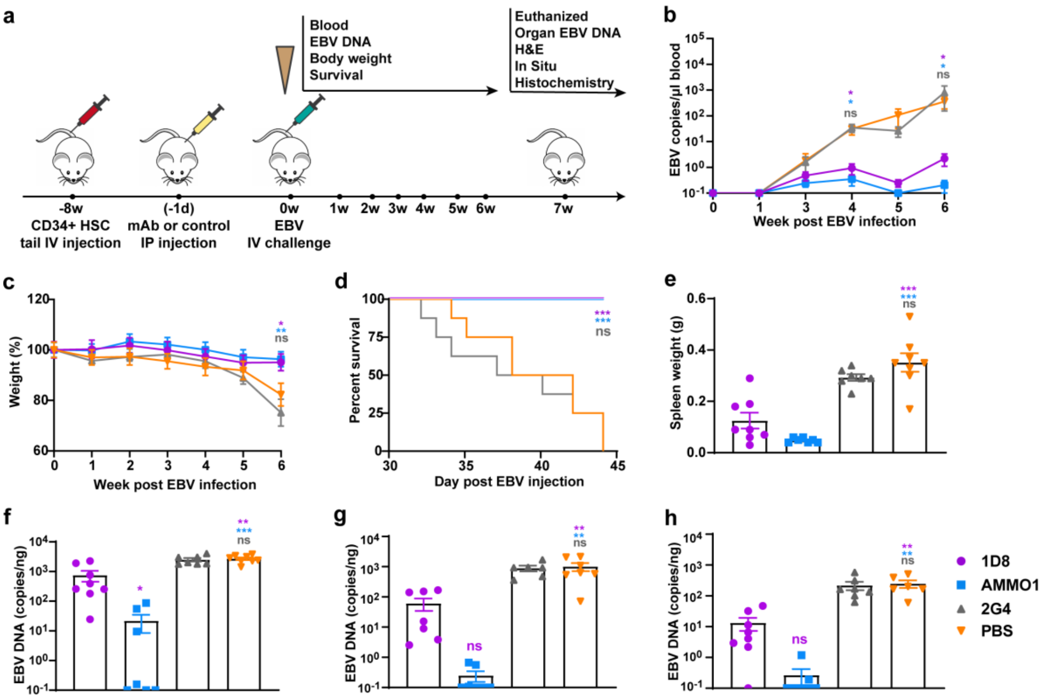

Supplementary Figure 2. Protective effect of single dose of 1D8 on fatal EBV attack in humanized mice (**a**) Timeline for engrafting CD34+ human hematopoietic stem cells (HSC), antibody administration, viral challenge, and monitoring for various biological and clinical outcomes. 400 μg of 1D8 (n=8), positive control AMMO1 (n=7), negative control 2G4 (n=7), or PBS (n=8) were administered to the humanized mice via intraperitoneal injection 24 h prior to intravenous challenge with Akata EBV. (**b**) EBV DNA in the peripheral blood, (**c**) body weight, and (**d**) survival were monitored weekly. On week 7 post infection, (**e**) spleen weight (**f**) virus titers in spleen, (**g**) liver, (**h**) kidney were analyzed. All data are presented as mean ± SEM. **p* < 0.05; ***p* < 0.01; ****p* < 0.001; ns, no significant, two-tailed unpaired Student's *t*-test. (**b**) 1D8 vs PBS in 4w **p*=0.038, AMMO1 vs PBS in 4w **p*=0.048, 1D8 vs PBS in 6w **p*=0.011, AMMO1 vs PBS in 6w **p*=0.017; (**c**) **p*=0.016, ***p*=0.009; (**d**) ****p* < 0.001, log-rank test (Mantel-Cox); (**e**) 1D8 vs PBS ****p*=0.0003, AMMO1 vs PBS ****p* < 0.001; (**f**) ***p*=0.0018, ****p* < 0.001; **p*=0.0417; (**g**) 1D8 vs PBS ***p*=0.005, AMMO1 vs PBS ***p*=0.0057; (**h**) 1D8 vs PBS ***p*=0.0017, AMMO1 vs PBS ***p*=0.0022. Source data are provided as a Source Data File.

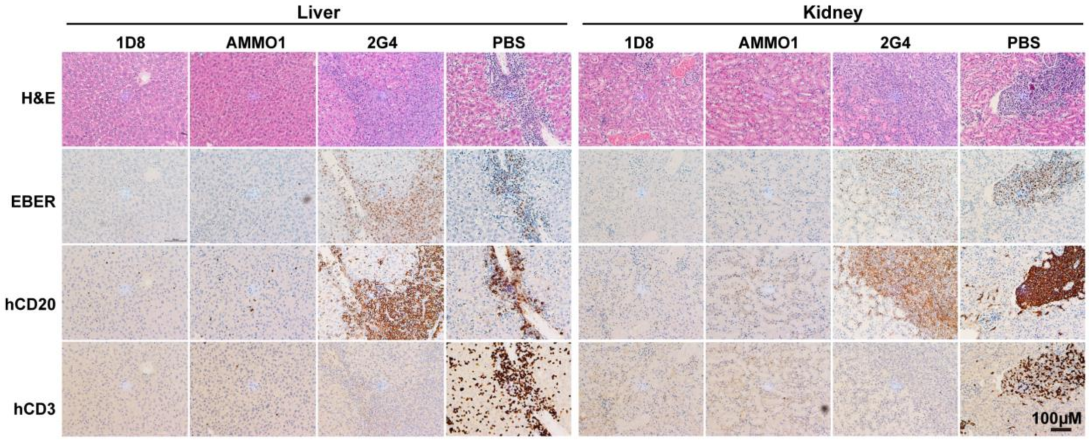

Supplementary Figure 3. 1D8 reduces viral replication and tissue damages in liver and kidney of mice.

Hepatic and renal sections stained for hematoxylin and eosin (H&E), human CD20 (hCD20), human CD3 (hCD3), and EBV encoded RNA (EBER) at necropsy. Scale bar of 100 μm is shown. Each image is representative of a group of 7-8 mice.

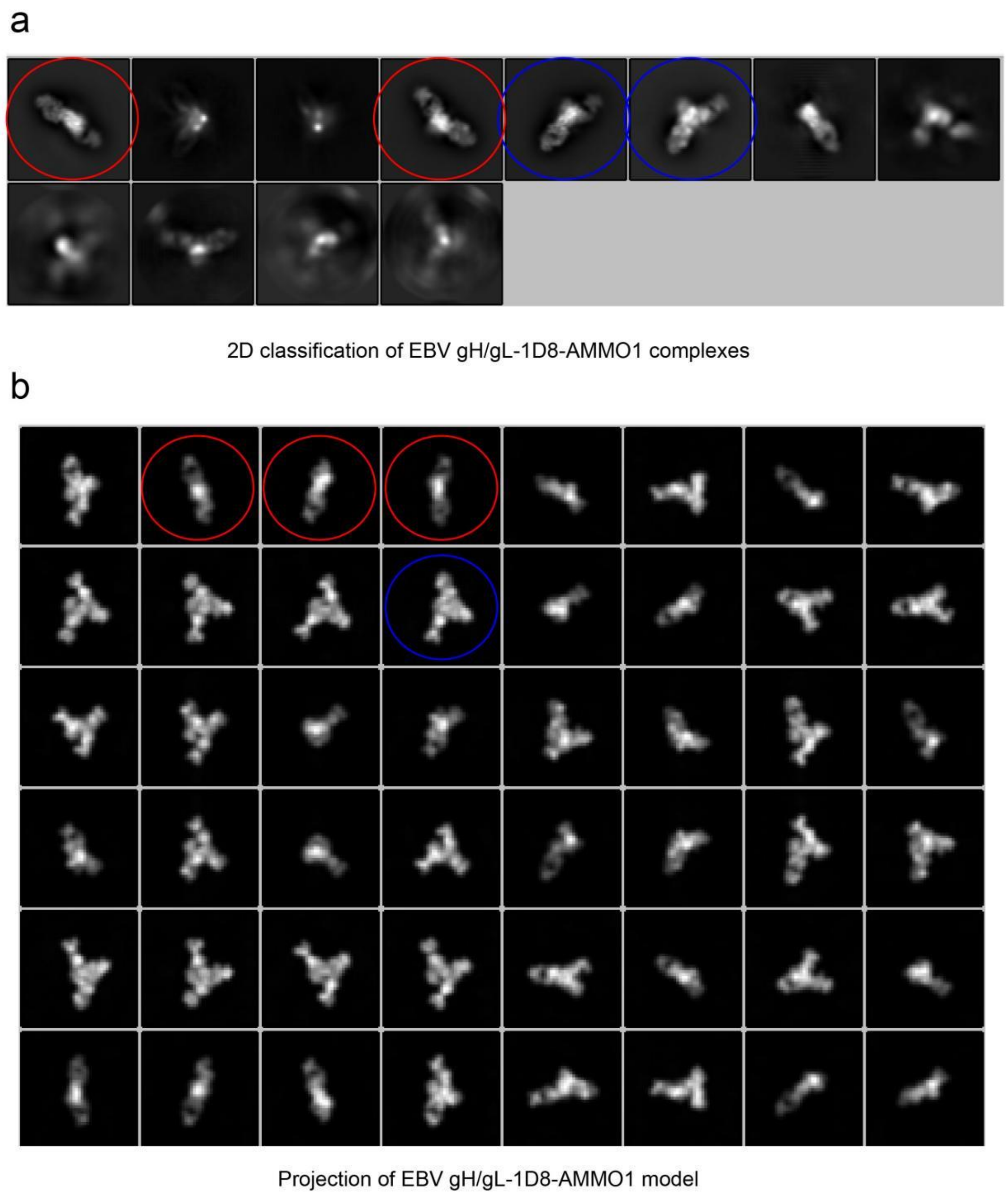

Supplementary Figure 4. The 2D classification results of gH/gL-1D8-AMMO1 ternary complex and the projection of merged gH/gL-1D8-AMMO1. (a) The 2D classification results of gH/gL-1D8-AMMO1 ternary complex. (b) The projection of merged gH/gL-1D8-AMMO1 model. Circles of the same color indicate projections of the same or similar orientation.

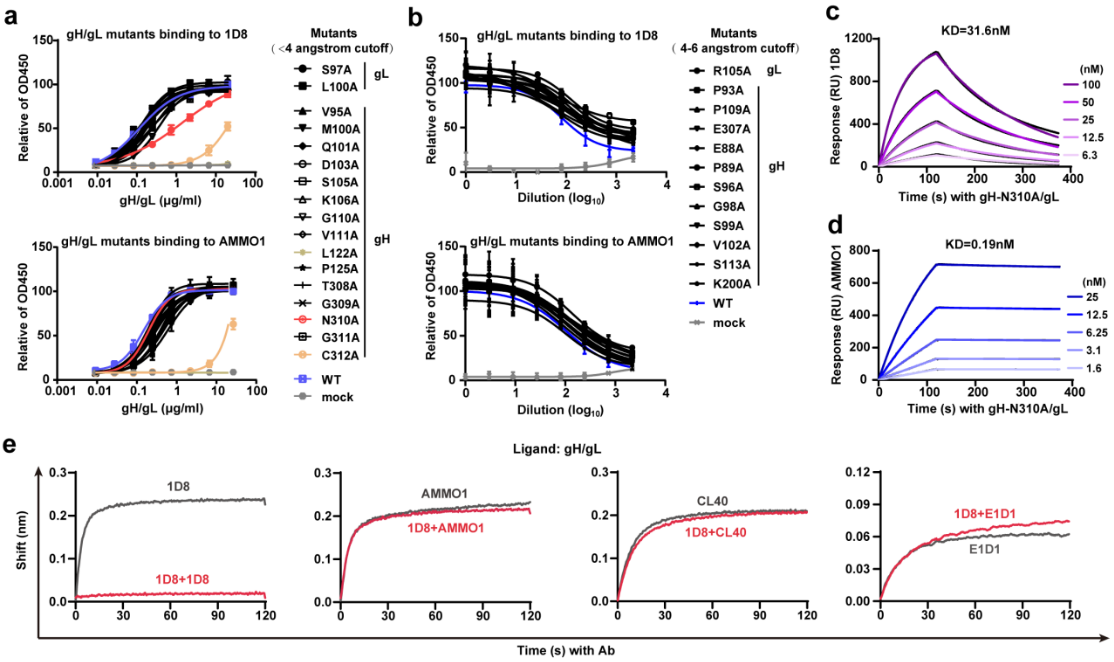

Supplementary Figure 5. Binding of 1D8 to gH/gL mutants and its competition with other gH/gL antibodies.

1D8 and AMMO1 binding to various gH/gL mutants measured by ELISA, determined by using a 4 angstrom (a) and 4-6 angstrom (b) distance cutoff. ELISA was performed in duplicate wells, and the data shown are means with SEM from two replicates. Binding activity of 1D8 (**c**) and AMMO1 (**d**) to gH-N310A/gL mutant measured by SPR. (**e**) Competitive binding of 1D8 with AMMO1, CL40 or E1D1 to gH/gL measured by BLI. Source data are provided as a Source Data File.

Supplementary Tables

Supplementary Table 1. Neutralization potency of monoclonal antibodies.

Mab	IC_{50} (μg/ml)			
	HNE1	Raji	Primary B cells	NPEC1-Bmi1
1D8	0.123	0.238	0.361	0.173
2A6	0.745	1.320	-	-
AMMO1	0.127	0.318	0.227	~10
2G4	NA	NA	NA	NA

Supplementary Table 2. Kinetic Analysis of Antibodies Binding to gH/gL Measured by SPR.

Ligand	Anylate	kon (1/Ms) ×10^5	koff (1/s) ×10^{-4}	KD (nM)
1D8	gH/gL S97A	1.33	1.36	1.02
1D8	gH/gL L100A	1.07	1.55	1.44
1D8	gH V95A/gL	1.30	2.31	1.77
1D8	gH M100A/gL	1.51	1.96	1.30
1D8	gH Q101A/gL	0.95	1.95	2.03
1D8	gH D103A/gL	0.72	0.69	0.95
1D8	gH S105A/gL	0.90	0.97	1.08
1D8	gH K106A/gL	1.26	1.09	0.86
1D8	gH G110A/gL	2.66	0.26	0.10
1D8	gH V111A/gL	1.11	1.48	1.34
1D8	gH P125A/gL	0.71	2.00	2.79
1D8	gH T308A/gL	1.04	1.01	0.97
1D8	gH G309A/gL	0.95	0.95	1.00
1D8	gH N310A/gL	1.75	55.20	31.60
1D8	gH G311A/gL	0.78	2.05	2.62
1D8	gH/gL WT	2.45	1.46	0.59
AMMO1	gH N310A/gL	4.97	0.96	0.19
AMMO1	gH/gL WT	5.77	0.85	0.14

Supplementary Table 3. Data collection and refinement statistics.

	EBV gH/gL-1D8
Data collection	
Space group	$P4_12_12$
Cell dimensions	
a, *b*, *c* (Å)	212.865, 212.865, 598.128
α, β, γ, (°)	90, 90, 90
Resolution (Å)	50.03-4.201(4.351-4.201) *
R_{sym} or R_{merge}	0.131 (1.261)
I / sI	8 (1.4)
Completeness (%)	99.34 (98.99)
Redundancy	8.1 (8.2)
Refinement	
Resolution (Å)	50.03-4.201
No. reflections	100335
R_{work} / R_{free}	25.30/28.33
No. atoms	
Protein	36304
B-factors	
Protein	153.13
R.m.s. deviations	
Bond lengths (Å)	0.004
Bond angles (°)	0.81
Ramachandran plot (%)	
Favored	93.35%
Allowed	6.54%
outlier	0.11%

One crystal was used.
*Values in parentheses are for highest-resolution shell.
