# Supplementary material for: A potent and protective human neutralizing antibody targeting a novel vulnerable site of Epstein-Barr virus
Source: Nat Commun. 2021 Nov 16;12:6624. doi: 10.1038/s41467-021-26912-6 (PMC8595662; doi:10.1038/s41467-021-26912-6)
Supplement: Supplementary file 2 — Reporting Summary [file 41467_2021_26912_MOESM2_ESM.pdf]

nature research

Corresponding author(s): Linqi Zhang
Last updated by author(s): Oct 19, 2021

Reporting Summary

Nature Research wishes to improve the reproducibility of the work that we publish. This form provides structure for consistency and transparency in reporting. For further information on Nature Research policies, see our Editorial Policies and the Editorial Policy Checklist.

Statistics

For all statistical analyses, confirm that the following items are present in the figure legend, table legend, main text, or Methods section.

n/a	Confirmed	
☐	☒	The exact sample size (*n*) for each experimental group/condition, given as a discrete number and unit of measurement
☐	☒	A statement on whether measurements were taken from distinct samples or whether the same sample was measured repeatedly
☐	☒	The statistical test(s) used AND whether they are one- or two-sided<br>*Only common tests should be described solely by name; describe more complex techniques in the Methods section.*
☒	☐	A description of all covariates tested
☒	☐	A description of any assumptions or corrections, such as tests of normality and adjustment for multiple comparisons
☐	☒	A full description of the statistical parameters including central tendency (e.g. means) or other basic estimates (e.g. regression coefficient) AND variation (e.g. standard deviation) or associated estimates of uncertainty (e.g. confidence intervals)
☐	☒	For null hypothesis testing, the test statistic (e.g. *F*, *t*, *r*) with confidence intervals, effect sizes, degrees of freedom and *P* value noted<br>*Give P values as exact values whenever suitable.*
☒	☐	For Bayesian analysis, information on the choice of priors and Markov chain Monte Carlo settings
☒	☐	For hierarchical and complex designs, identification of the appropriate level for tests and full reporting of outcomes
☒	☐	Estimates of effect sizes (e.g. Cohen's *d*, Pearson's *r*), indicating how they were calculated

Our web collection on statistics for biologists contains articles on many of the points above.

Software and code

Policy information about availability of computer code

Data collection	Flow cytometry BD Aria II was used for for cell sorting and Biacore 8K Control Software was used for binding kinetic studies.
Data analysis	Graphs were presented by GraphPad Prism version 8 and Biacore Insight Evaluation Software version 3.0.12.15655. Flow cytometry data analysis was performed using FlowJo version 10 software. HKL2000 was used for crystal data processing. RELION 3.1 was used for cryo-EM data processing. PHASER (CCP4 Program Suite) was used for molecular replacement to solve the complex structure. COOT was used for model building and refinement manually. Phenix was used for automatical model refinement.

For manuscripts utilizing custom algorithms or software that are central to the research but not yet described in published literature, software must be made available to editors and reviewers. We strongly encourage code deposition in a community repository (e.g. GitHub). See the Nature Research guidelines for submitting code & software for further information.

Data

Policy information about availability of data

All manuscripts must include a data availability statement. This statement should provide the following information, where applicable:

- Accession codes, unique identifiers, or web links for publicly available datasets
- A list of figures that have associated raw data
- A description of any restrictions on data availability

Data generated or analyzed during this study are included in this published article (and its supplementary information files). All other data are also available from the corresponding author upon reasonable requests. The atomic model of 1D8-gH/gL (PDB ID: 7D5Z) has been deposited in the Protein Data Bank. Source data are provided with this paper.

Field-specific reporting

Please select the one below that is the best fit for your research. If you are not sure, read the appropriate sections before making your selection.

☒ Life sciences ☐ Behavioural & social sciences ☐ Ecological, evolutionary & environmental sciences

For a reference copy of the document with all sections, see nature.com/documents/nr-reporting-summary-flat.pdf

Life sciences study design

All studies must disclose on these points even when the disclosure is negative.

Sample size	No sample-size calculation was performed. The number of mice in vivo protection assay in each group was 7-8, which is acceptable in the field.
Data exclusions	No data were excluded.
Replication	ELISA analysis, neutralization assay, epitope mapping experiments, mabs binding to cell surface expressed gH/gL protein were peformed two times independently. EBV DNA detections in blood and tissue were performed three times. Antibodies blocking in viral fusion and binding were performed three times. SPR assays for antibody kinetics were performed twice. BLI assays for antibody blocking in antigen-receptor binding were performed twice. All attempts at replication were successful.
Randomization	Not applicable for this study as no treatment strategies are compared.
Blinding	No blinding was conducted since there was no specific grouping.

Behavioural & social sciences study design

All studies must disclose on these points even when the disclosure is negative.

Study description	*Briefly describe the study type including whether data are quantitative, qualitative, or mixed-methods (e.g. qualitative cross-sectional, quantitative experimental, mixed-methods case study).*
Research sample	*State the research sample (e.g. Harvard university undergraduates, villagers in rural India) and provide relevant demographic information (e.g. age, sex) and indicate whether the sample is representative. Provide a rationale for the study sample chosen. For studies involving existing datasets, please describe the dataset and source.*
Sampling strategy	*Describe the sampling procedure (e.g. random, snowball, stratified, convenience). Describe the statistical methods that were used to predetermine sample size OR if no sample-size calculation was performed, describe how sample sizes were chosen and provide a rationale for why these sample sizes are sufficient. For qualitative data, please indicate whether data saturation was considered, and what criteria were used to decide that no further sampling was needed.*
Data collection	*Provide details about the data collection procedure, including the instruments or devices used to record the data (e.g. pen and paper, computer, eye tracker, video or audio equipment) whether anyone was present besides the participant(s) and the researcher, and whether the researcher was blind to experimental condition and/or the study hypothesis during data collection.*
Timing	*Indicate the start and stop dates of data collection. If there is a gap between collection periods, state the dates for each sample cohort.*
Data exclusions	*If no data were excluded from the analyses, state so OR if data were excluded, provide the exact number of exclusions and the rationale behind them, indicating whether exclusion criteria were pre-established.*
Non-participation	*State how many participants dropped out/declined participation and the reason(s) given OR provide response rate OR state that no participants dropped out/declined participation.*
Randomization	*If participants were not allocated into experimental groups, state so OR describe how participants were allocated to groups, and if allocation was not random, describe how covariates were controlled.*

Ecological, evolutionary & environmental sciences study design

All studies must disclose on these points even when the disclosure is negative.

Study description	*Briefly describe the study. For quantitative data include treatment factors and interactions, design structure (e.g. factorial, nested, hierarchical), nature and number of experimental units and replicates.*

Research sample	*Describe the research sample (e.g. a group of tagged Passer domesticus, all Stenocereus thurberi within Organ Pipe Cactus National Monument), and provide a rationale for the sample choice. When relevant, describe the organism taxa, source, sex, age range and any manipulations. State what population the sample is meant to represent when applicable. For studies involving existing datasets, describe the data and its source.*
Sampling strategy	*Note the sampling procedure. Describe the statistical methods that were used to predetermine sample size OR if no sample-size calculation was performed, describe how sample sizes were chosen and provide a rationale for why these sample sizes are sufficient.*
Data collection	*Describe the data collection procedure, including who recorded the data and how.*
Timing and spatial scale	*Indicate the start and stop dates of data collection, noting the frequency and periodicity of sampling and providing a rationale for these choices. If there is a gap between collection periods, state the dates for each sample cohort. Specify the spatial scale from which the data are taken*
Data exclusions	*If no data were excluded from the analyses, state so OR if data were excluded, describe the exclusions and the rationale behind them, indicating whether exclusion criteria were pre-established.*
Reproducibility	*Describe the measures taken to verify the reproducibility of experimental findings. For each experiment, note whether any attempts to repeat the experiment failed OR state that all attempts to repeat the experiment were successful.*
Randomization	*Describe how samples/organisms/participants were allocated into groups. If allocation was not random, describe how covariates were controlled. If this is not relevant to your study, explain why.*
Blinding	*Describe the extent of blinding used during data acquisition and analysis. If blinding was not possible, describe why OR explain why blinding was not relevant to your study.*

Did the study involve field work? ☐ Yes ☐ No

Field work, collection and transport

Field conditions	*Describe the study conditions for field work, providing relevant parameters (e.g. temperature, rainfall).*
Location	*State the location of the sampling or experiment, providing relevant parameters (e.g. latitude and longitude, elevation, water depth).*
Access & import/export	*Describe the efforts you have made to access habitats and to collect and import/export your samples in a responsible manner and in compliance with local, national and international laws, noting any permits that were obtained (give the name of the issuing authority, the date of issue, and any identifying information).*
Disturbance	*Describe any disturbance caused by the study and how it was minimized.*

Reporting for specific materials, systems and methods

We require information from authors about some types of materials, experimental systems and methods used in many studies. Here, indicate whether each material, system or method listed is relevant to your study. If you are not sure if a list item applies to your research, read the appropriate section before selecting a response.

Materials & experimental systems

n/a	Involved in the study	
☐	☒	Antibodies
☐	☒	Eukaryotic cell lines
☒	☐	Palaeontology and archaeology
☐	☒	Animals and other organisms
☐	☒	Human research participants
☒	☐	Clinical data
☒	☐	Dual use research of concern

Methods

n/a	Involved in the study	
☒	☐	ChIP-seq
☐	☒	Flow cytometry
☒	☐	MRI-based neuroimaging

Antibodies

Antibodies used	AFor identification of human specific memory B cells for production of monoclonal antibodies, CD20-PE-Cy7 (PE-Cy™7 Mouse Anti-Human CD20, BD Pharmingen, cat. 335793, clone L27, lot. 3129950, 1:50 dilution), CD3-PE-Cy5 (PE-Cy5™ Mouse Anti-Human CD3, BD Pharmingen, cat. 555341, clone HIT3a, lot.81339, 1:12.5 dilution), CD19-APC-Cy7 (APC-Cy7™ Mouse Anti-Human CD19, BD Pharmingen, cat. 348794, clone SJ25C1, lot.9239271, 1:50 dilution), CD14-PE-Cy5 (PE-Cy5™ Mouse Anti-Human CD14, eBioscience, cat. 15-0149-42, clone 61D3, lot. 2178335, 1:25 dilution), CD235a-PE-Cy5 (PE-Cy5™ Mouse Anti-Human CD235a, BD Pharmingen, cat. 559944, clone GA-R2, lot. 3228551, 1:25dilution), CD16-PE-Cy5 (PE-Cy™5 Mouse Anti-Human CD16, BD Pharmingen, cat.555408, clone 3G8, Lot.2117564, 1:12.5 dilution), IgG-FITC (FITC Mouse Anti-Human IgG, BD Pharmingen, cat. 555786, clone G18-145, lot. 8284569, 1:12.5 dilution), anti-his-PE (PE anti-His Tag Antibody, BioLegend, cat. 362603, clone J095G46, 1:100 dilution) antibodies were used.

For characterization of human antibodies, secondary anti-human IgG-HRP (HRP goat anti-human IgG (H+L), Promega, cat. W4031, polyclonal, lot.0000297692, 1:5000 dilution) and anti-human IgG-PerCP-Cy5.5 (PerCP/Cyanine5.5 anti-human IgG Fc Antibody, Biolegend, cat. 409312, clone HP6017, 1:100 dilution) antibodies were used.
For Detection of human immune cells in the blood of humanized mice, CD45-PE (PE Mouse Anti-Human CD45, BD Pharmingen, cat. 555483, clone HI30, 1:100 dilution), CD20-FITC (FITC Mouse Anti-Human CD20, BD Pharmingen, cat. 555622, clone 2H7, 1:100 dilution), CD3-PerCP-Cy5.5 (PerCP-Cy™5.5 Mouse Anti-Human CD3, BD Pharmingen, cat. 560835, clone UCHT1, 1:100 dilution) antibodies were used.
For the binding analysis of gH/gL mutants, mouse anti-his antibody (ProteinFind® Anti-His Mouse Monoclonal Antibody, TRANSGEN BIOTECH, cat. HT501-02, 1:3000 dilution) and goat anti-mouse-HRP antibody (Goat anti-Mouse IgG (H+L) Secondary Antibody, HRP, Invitrogen, Cat. 31430, Lot. 0000169565, 1:5000 dilution) antibodies were used.
For Immunostaining of human T cells and B cells in tissues, rabbit anti-human CD20 (anti-CD20 primary antibody, VENTANA. cat. 760-2531, Lot. F22805, 1:200 dilution) rabbit anti-human CD3 (anti-CD3 primary antibody, VENTANA. cat. 790-4341, Lot. F16525, 1:200 dilution) and goat anti-rabbit HRP (Goat anti-Rabbit IgG (H+L) Secondary Antibody, HRP, Invitrogen, Cat. 31460, 1:5000 dilution) antibodies were used.
anti-EBV 1D8 was produced in our lab using 293F cells and stocked as 1mg/ml in PBS buffer (Lot#20190801); Anti-EBV AMMO1 was produced in our lab using 293F cells and stocked as 1mg/ml in PBS buffer (Lot#20190807); Anti-Ebola 2G4 was produced in our lab using 293F cells and stocked as 1mg/ml in PBS buffer (Lot#20190805); Anti-EBV CL40,E1D1,CL59 were produced in our lab using 293F cells and stocked as 1mg/ml in PBS buffer,respectively (Lot#20190901, Lot#20190902,Lot#20190903).

Validation

All the antibodies used in this study were commercial antibodies and were only used for applications, with validation procedures described on the following sites of the manufacturers:
CD20-PE-Cy7 (PE-Cy™7 Mouse Anti-Human CD20, BD Pharmingen, cat. 335793, clone L27, lot. 3129950, 1:50 dilution), https://www.bdbiosciences.com/cn/applications/research/stem-cell-research/hematopoietic-stem-cell-markers/human/negative-markers/pe-cytrade7-mouse-anti-human-cd20-l27/p/335793
CD3-PE-Cy5 (PE-Cy5™ Mouse Anti-Human CD3, BD Pharmingen, cat. 555341, clone HIT3a, lot.81339, 1:12.5 dilution), https://www.bdbiosciences.com/cn/applications/research/t-cell-immunology/th-1-cells/surface-markers/human/pe-cy5-mouse-anti-human-cd3-hit3a/p/555341
CD19-APC-Cy7 (APC-Cy7™ Mouse Anti-Human CD19, BD Pharmingen, cat. 348794, clone SJ25C1, lot.9239271, 1:50 dilution), https://www.bdbiosciences.com/cn/applications/research/stem-cell-research/hematopoietic-stem-cell-markers/human/negative-markers/apc-cytrade7-mouse-anti-human-cd19-sj25c1-also-known-as-sj25-c1/p/348794
CD14-PE-Cy5 (PE-Cy5™ Mouse Anti-Human CD14, eBioscience, cat. 15-0149-42, clone 61D3, lot. 2178335, 1:25 dilution), https://www.thermofisher.com/cn/zh/antibody/product/CD14-Antibody-clone-61D3-Monoclonal/15-0149-42
CD235a-PE-Cy5 (PE-Cy5™ Mouse Anti-Human CD235a, BD Pharmingen, cat. 559944, clone GA-R2, lot. 3228551, 1:25dilution), https://www.bdbiosciences.com/cn/reagents/research/antibodies-buffers/immunology-reagents/anti-human-antibodies/cell-surface-antigens/pe-cy5-mouse-anti-human-cd235a-ga-r2-hir2/p/559944
CD16-PE-Cy5 (PE-Cy™5 Mouse Anti-Human CD16, BD Pharmingen, cat.555408, clone 3G8, Lot.2117564, 1:12.5 dilution)https://www.bdbiosciences.com/cn/applications/research/stem-cell-research/cancer-research/human/pe-cy5-mouse-anti-human-cd16-3g8/p/555408
IgG-FITC (FITC Mouse Anti-Human IgG, BD Pharmingen, cat. 555786, clone G18-145, lot. 8284569, 1:12.5 dilution), https://www.bdbiosciences.com/cn/applications/research/b-cell-research/immunoglobulins/human/fitc-mouse-anti-human-igg-g18-145/p/555786
anti-his-PE (PE anti-His Tag Antibody, BioLegend, cat. 362603, clone J095G46, 1:100 dilution) , https://www.biolegend.com/en-us/products/pe-anti-his-tag-antibody-9861
secondary anti-human IgG-HRP (HRP goat anti-human IgG (H+L), Promega, cat. W4031, polyclonal, lot.0000297692, 1:5000 dilution)
anti-human IgG-PerCP-Cy5.5 (PerCP/Cyanine5.5 anti-human IgG Fc Antibody, Biolegend, cat. 409312, clone HP6017, 1:100 dilution)
https://www.biolegend.com/en-us/products/percp-cyanine5-5-anti-human-igg-fc-8392
CD45-PE (PE Mouse Anti-Human CD45, BD Pharmingen, cat. 555483, clone HI30, 1:100 dilution)
https://www.bdbiosciences.com/cn/applications/research/stem-cell-research/cancer-research/human/pe-mouse-anti-human-cd45-hi30/p/555483
CD20-FITC (FITC Mouse Anti-Human CD20, BD Pharmingen, cat. 555622, clone 2H7, 1:100 dilution)
https://www.bdbiosciences.com/cn/applications/research/stem-cell-research/hematopoietic-stem-cell-markers/human/negative-markers/fitc-mouse-anti-human-cd20-2h7/p/555622
CD3-PerCP-Cy5.5 (PerCP-Cy™5.5 Mouse Anti-Human CD3, BD Pharmingen, cat. 560835, clone UCHT1, 1:100 dilution)
https://www.bdbiosciences.com/cn/applications/research/t-cell-immunology/th-1-cells/surface-markers/human/percp-cy55-mouse-anti-human-cd3-ucht1-also-known-as-ucht-1-ucht-1/p/560835
Mouse anti-his antibody (ProteinFind® Anti-His Mouse Monoclonal Antibody, TRANSGEN BIOTECH, cat. HT501-02, 1:3000 dilution)
https://www.transgen.com.cn/antibody_tag/385.html
goat anti-mouse-HRP antibody (Goat anti-Mouse IgG (H+L) Secondary Antibody, HRP, Invitrogen, Cat. 31430, Lot. 0000169565, 1:5000 dilution)
https://www.thermofisher.com/cn/zh/antibody/product/Goat-anti-Mouse-IgG-H-L-Secondary-Antibody-Polyclonal/31430
goat anti-rabbit HRP (Goat anti-Rabbit IgG (H+L) Secondary Antibody, HRP, Invitrogen, Cat. 31460, 1:5000 dilution)
https://www.thermofisher.com/cn/zh/antibody/product/Goat-anti-Rabbit-IgG-H-L-Secondary-Antibody-Polyclonal/31460Antibodies 1D8 and AMMO1 were isolated from human, 2G4, CL40, CL59 and E1D1 were isolated from mice. The VH and VK/Vλ genes of these antibodies were obtained from PDB and codon optimized genes were synthesized by Tsingke Biological Technology Company. The PCR product of these antibodies were cloned into antibody expression vectors containing the constant regions of human IgG1.

Eukaryotic cell lines

Policy information about cell lines

Cell line source(s)	The 293T cells, CHO-K1 cells, and Raji cells were obtained from ATCC. The 293F cells were purchased from ThermoFisher. The HNE1 cells and HK1 cells were a kind gift from Professor Sai-Wah Tsao (University of Hong Kong, Hong Kong SAR); EBV-positive Akata cells were a kind gift from Professor Maria G. Masucci (Karolinska Institute, Sweden).Bmi1-immortalized nasopharyngeal epithelial cell line (NPEC1-Bmi1) was established in our laboratory.
Authentication	All cell lines were frequently checked for cellular morphologies, growth rates and functions.
Mycoplasma contamination	We confirm that all cell lines were negative for mycoplasma contamination.
Commonly misidentified lines (See ICLAC register)	No commonly misidentified cell lines were used.

Palaeontology and Archaeology

Specimen provenance	*Provide provenance information for specimens and describe permits that were obtained for the work (including the name of the issuing authority, the date of issue, and any identifying information).*
Specimen deposition	*Indicate where the specimens have been deposited to permit free access by other researchers.*
Dating methods	*If new dates are provided, describe how they were obtained (e.g. collection, storage, sample pretreatment and measurement), where they were obtained (i.e. lab name), the calibration program and the protocol for quality assurance OR state that no new dates are provided.*

☐ Tick this box to confirm that the raw and calibrated dates are available in the paper or in Supplementary Information.

Ethics oversight	*Identify the organization(s) that approved or provided guidance on the study protocol, OR state that no ethical approval or guidance was required and explain why not.*

Note that full information on the approval of the study protocol must also be provided in the manuscript.

Animals and other organisms

Policy information about studies involving animals; ARRIVE guidelines recommended for reporting animal research

Laboratory animals	The humanized mice was based on NOD.Cg-Prkdcem1IDMOIl2rgem2IDMO mice (NOD-Prkdcnull IL2Rγnull, NPI®), which were kept in a specific pathogen free (SPF) facility and obtained from BEIJING IDMO Co., Ltd. All the humanized mice were female and 12 weeks old. Sixty mice were used in the study.
Wild animals	The study did not involve wild animals.
Field-collected samples	The study did not involve field-collected samples.
Ethics oversight	This study was reviewed and approved by the Ethics Committee of the Sun Yat-Sen University Cancer Center.

Note that full information on the approval of the study protocol must also be provided in the manuscript.

Human research participants

Policy information about studies involving human research participants

Population characteristics	We collected plasma samples from 48 participants including 23 histologically diagnosed NPC cases and 25 non-NPC high-risk healthy controls from 2007 and 2018, including 25 males and 23 females, aging 30-59 years old. There are Peripheral blood mononuclear cell (PBMC) sample of donors 27 were collected in 2018. We collected PBMC samples from two healthy donors and isolated primary B cells.
Recruitment	Study participants were recruited in a screening program in Sihui County in Guangdong Province of China. There was no potential self-selection bias or other biases during the selection. In order to maximize the participation rate, members of the target populations were contacted by their village doctors who introduced the screening program with information leaflets containing information on NPC in general (such as possible causes of NPC, early symptoms, diagnosis, treatment, and prognosis), the advantages and potential side effects of the screening method, and the follow-up strategies in case of a positive test result. The screening was also promoted by local television stations in the screening towns. This mass screening study (NCT00941538 Clinical Trials.gov) was approved by the Ethics Review Committee of the Sun Yat-sen University Cancer Center (SYSUCC, YP2009051).
Ethics oversight	This study was reviewed and approved by the Ethics Committee of the Sun Yat-Sen University Cancer Center.

Note that full information on the approval of the study protocol must also be provided in the manuscript.

Clinical data

Policy information about clinical studies

All manuscripts should comply with the ICMJE guidelines for publication of clinical research and a completed CONSORT checklist must be included with all submissions.

Clinical trial registration	*Provide the trial registration number from ClinicalTrials.gov or an equivalent agency.*
Study protocol	*Note where the full trial protocol can be accessed OR if not available, explain why.*
Data collection	*Describe the settings and locales of data collection, noting the time periods of recruitment and data collection.*
Outcomes	*Describe how you pre-defined primary and secondary outcome measures and how you assessed these measures.*

Dual use research of concern

Policy information about dual use research of concern

Hazards

Could the accidental, deliberate or reckless misuse of agents or technologies generated in the work, or the application of information presented in the manuscript, pose a threat to:

No	Yes	
☐	☐	Public health
☐	☐	National security
☐	☐	Crops and/or livestock
☐	☐	Ecosystems
☐	☐	Any other significant area

Experiments of concern

Does the work involve any of these experiments of concern:

No	Yes	
☐	☐	Demonstrate how to render a vaccine ineffective
☐	☐	Confer resistance to therapeutically useful antibiotics or antiviral agents
☐	☐	Enhance the virulence of a pathogen or render a nonpathogen virulent
☐	☐	Increase transmissibility of a pathogen
☐	☐	Alter the host range of a pathogen
☐	☐	Enable evasion of diagnostic/detection modalities
☐	☐	Enable the weaponization of a biological agent or toxin
☐	☐	Any other potentially harmful combination of experiments and agents

ChIP-seq

Data deposition

☐ Confirm that both raw and final processed data have been deposited in a public database such as GEO.

☐ Confirm that you have deposited or provided access to graph files (e.g. BED files) for the called peaks.

Data access links<br>*May remain private before publication.*	*For "Initial submission" or "Revised version" documents, provide reviewer access links. For your "Final submission" document, provide a link to the deposited data.*
Files in database submission	*Provide a list of all files available in the database submission.*
Genome browser session<br>(e.g. UCSC)	*Provide a link to an anonymized genome browser session for "Initial submission" and "Revised version" documents only, to enable peer review. Write "no longer applicable" for "Final submission" documents.*

Methodology

Replicates	*Describe the experimental replicates, specifying number, type and replicate agreement.*
Sequencing depth	*Describe the sequencing depth for each experiment, providing the total number of reads, uniquely mapped reads, length of reads and whether they were paired- or single-end.*

Antibodies	*Describe the antibodies used for the ChIP-seq experiments; as applicable, provide supplier name, catalog number, clone name, and lot number.*
Peak calling parameters	*Specify the command line program and parameters used for read mapping and peak calling, including the ChIP, control and index files used.*
Data quality	*Describe the methods used to ensure data quality in full detail, including how many peaks are at FDR 5% and above 5-fold enrichment.*
Software	*Describe the software used to collect and analyze the ChIP-seq data. For custom code that has been deposited into a community repository, provide accession details.*

Flow Cytometry

Plots

Confirm that:

- [x] The axis labels state the marker and fluorochrome used (e.g. CD4-FITC).
- [x] The axis scales are clearly visible. Include numbers along axes only for bottom left plot of group (a 'group' is an analysis of identical markers).
- [x] All plots are contour plots with outliers or pseudocolor plots.
- [x] A numerical value for number of cells or percentage (with statistics) is provided.

Methodology

Sample preparation	PBMCs from donors were collected and incubated with an antibody and gH/gL for identification of gH/Gl specific B cells. The cocktail consisted of CD20-PE-Cy7, CD19-APC-Cy7, CD3-PE-Cy5, CD16-PE-Cy5, CD235a-PE-Cy5, IgG-FITC (BD Biosciences), CD14-PE-Cy5 (eBioscience) and the recombinant gH/gL-His. Two consecutive staining steps were conducted. The first utilized an antibody and gH/gL-His for 1h at 4 °C. The second staining at 4 °C for 30min involved anti-his-PE antibodies (BioLegend) to target the His tag of gH/gL. The stained cells were washed and resuspended in PBS before being strained through a 70µm cell mesh (BD Biosciences). More information available on Methods sections.
Instrument	BD Aria II
Software	FlowJo version 10
Cell population abundance	The gH/gL-specific B cells constitute about 0.5% among the IgG+ B cell population.
Gating strategy	gH/gL-specific B cells were gated as CD19+CD20+CD3-CD16-CD14-CD235a-IgG+ gH/gL +. More Information available on Figure1a and methods sections.

- [x] Tick this box to confirm that a figure exemplifying the gating strategy is provided in the Supplementary Information.

Magnetic resonance imaging

Experimental design

Design type	*Indicate task or resting state; event-related or block design.*
Design specifications	*Specify the number of blocks, trials or experimental units per session and/or subject, and specify the length of each trial or block (if trials are blocked) and interval between trials.*
Behavioral performance measures	*State number and/or type of variables recorded (e.g. correct button press, response time) and what statistics were used to establish that the subjects were performing the task as expected (e.g. mean, range, and/or standard deviation across subjects).*

Acquisition

Imaging type(s)	*Specify: functional, structural, diffusion, perfusion.*
Field strength	*Specify in Tesla*
Sequence & imaging parameters	*Specify the pulse sequence type (gradient echo, spin echo, etc.), imaging type (EPI, spiral, etc.), field of view, matrix size, slice thickness, orientation and TE/TR/flip angle.*
Area of acquisition	*State whether a whole brain scan was used OR define the area of acquisition, describing how the region was determined.*

Diffusion MRI ☐ Used ☐ Not used

Preprocessing

Preprocessing software	*Provide detail on software version and revision number and on specific parameters (model/functions, brain extraction, segmentation, smoothing kernel size, etc.).*
Normalization	*If data were normalized/standardized, describe the approach(es): specify linear or non-linear and define image types used for transformation OR indicate that data were not normalized and explain rationale for lack of normalization.*
Normalization template	*Describe the template used for normalization/transformation, specifying subject space or group standardized space (e.g. original Talairach, MNI305, ICBM152) OR indicate that the data were not normalized.*
Noise and artifact removal	*Describe your procedure(s) for artifact and structured noise removal, specifying motion parameters, tissue signals and physiological signals (heart rate, respiration).*
Volume censoring	*Define your software and/or method and criteria for volume censoring, and state the extent of such censoring.*

Statistical modeling & inference

Model type and settings	*Specify type (mass univariate, multivariate, RSA, predictive, etc.) and describe essential details of the model at the first and second levels (e.g. fixed, random or mixed effects; drift or auto-correlation).*
Effect(s) tested	*Define precise effect in terms of the task or stimulus conditions instead of psychological concepts and indicate whether ANOVA or factorial designs were used.*

Specify type of analysis: ☐ Whole brain ☐ ROI-based ☐ Both

Statistic type for inference (See Eklund et al. 2016)	*Specify voxel-wise or cluster-wise and report all relevant parameters for cluster-wise methods.*
Correction	*Describe the type of correction and how it is obtained for multiple comparisons (e.g. FWE, FDR, permutation or Monte Carlo).*

Models & analysis

n/a	Involved in the study
☐	☐ Functional and/or effective connectivity
☐	☐ Graph analysis
☐	☐ Multivariate modeling or predictive analysis

Functional and/or effective connectivity	*Report the measures of dependence used and the model details (e.g. Pearson correlation, partial correlation, mutual information).*
Graph analysis	*Report the dependent variable and connectivity measure, specifying weighted graph or binarized graph, subject- or group-level, and the global and/or node summaries used (e.g. clustering coefficient, efficiency, etc.).*
Multivariate modeling and predictive analysis	*Specify independent variables, features extraction and dimension reduction, model, training and evaluation metrics.*
